# SELENOP modifies sporadic colorectal carcinogenesis and WNT signaling activity through LRP5/6 interactions

**DOI:** 10.1172/JCI165988

**Published:** 2023-07-03

**Authors:** Jennifer M. Pilat, Rachel E. Brown, Zhengyi Chen, Nathaniel J. Berle, Adrian P. Othon, M. Kay Washington, Shruti A. Anant, Suguru Kurokawa, Victoria H. Ng, Joshua J. Thompson, Justin Jacobse, Jeremy A. Goettel, Ethan Lee, Yash A. Choksi, Ken S. Lau, Sarah P. Short, Christopher S. Williams

**Affiliations:** 1 Program in Cancer Biology,; 2 Medical Scientist Training Program, and; 3 Program in Chemical and Physical Biology, Vanderbilt University School of Medicine, Nashville, Tennessee, USA.; 4 Epithelial Biology Center, VUMC, Nashville, Tennessee, USA.; 5 Department of Medicine, Vanderbilt University Medical Center (VUMC), Nashville, Tennessee, USA.; 6 VERTICES Postbaccalaureate Research Education Program and; 7 Department of Pathology, Microbiology, and Immunology, Vanderbilt University School of Medicine, Nashville, Tennessee, USA.; 8 Vanderbilt University, Nashville, Tennessee, USA.; 9 Department of Pharmacy, Osaka Ohtani University, Tondabayashi, Osaka, Japan.; 10 Willem Alexander Children’s Hospital, Leiden University Medical Center, Leiden, Netherlands.; 11 Veterans Affairs Tennessee Valley Healthcare System, Nashville, Tennessee, USA.; 12 Center for Mucosal Inflammation and Cancer, VUMC, Nashville, Tennessee, USA.; 13 Department of Cell and Developmental Biology and; 14 Department of Pharmacology, Vanderbilt University School of Medicine, Nashville, Tennessee, USA.; 15 Department of Surgery, VUMC, Nashville, Tennessee, USA.; 16 Department of Internal Medicine, University of Iowa, Iowa City, Iowa, USA.

**Keywords:** Gastroenterology, Colorectal cancer

## Abstract

Although selenium deficiency correlates with colorectal cancer (CRC) risk, the roles of the selenium-rich antioxidant selenoprotein P (SELENOP) in CRC remain unclear. In this study, we defined SELENOP’s contributions to sporadic CRC. In human single-cell cRNA-Seq (scRNA-Seq) data sets, we discovered that *SELENOP* expression rose as normal colon stem cells transformed into adenomas that progressed into carcinomas. We next examined the effects of *Selenop* KO in a mouse adenoma model that involved conditional, intestinal epithelium-specific deletion of the tumor suppressor adenomatous polyposis coli (*Apc*) and found that *Selenop* KO decreased colon tumor incidence and size. We mechanistically interrogated SELENOP-driven phenotypes in tumor organoids as well as in CRC and noncancer cell lines. *Selenop*-KO tumor organoids demonstrated defects in organoid formation and decreases in WNT target gene expression, which could be reversed by SELENOP restoration. Moreover, SELENOP increased canonical WNT signaling activity in noncancer and CRC cell lines. In defining the mechanism of action of SELENOP, we mapped protein-protein interactions between SELENOP and the WNT coreceptors low-density lipoprotein receptor–related proteins 5 and 6 (LRP5/6). Last, we confirmed that SELENOP-LRP5/6 interactions contributed to the effects of SELENOP on WNT activity. Overall, our results position SELENOP as a modulator of the WNT signaling pathway in sporadic CRC.

## Graphical abstract

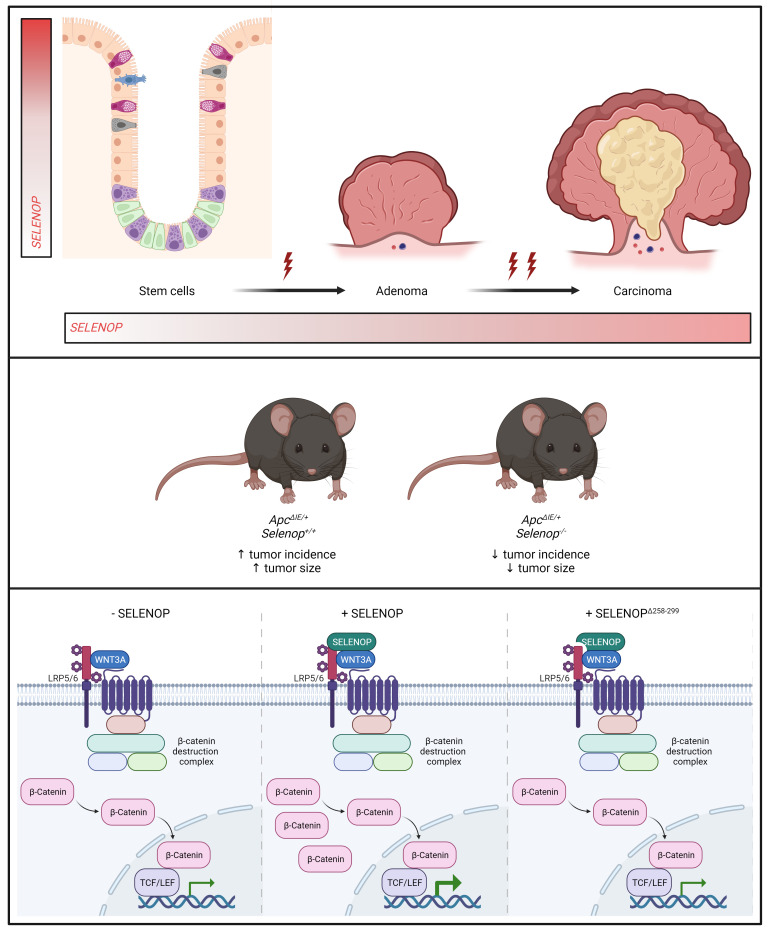

## Introduction

Both human observational and animal preclinical studies support tumor-protective roles for the micronutrient selenium in the gastrointestinal tract; however, human clinical trials have yet to corroborate these findings (1–7). Selenium is thought to exert its biological functions through incorporation into selenocysteine-containing proteins, or selenoproteins (8). Among the known selenoproteins, selenoprotein P (SELENOP) is unique in that it contains multiple selenocysteines: 1 selenocysteine in an N-terminal antioxidant domain and 9 selenocysteines in a C-terminal selenium transport domain. Although SELENOP is largely synthesized by the liver and secreted into the plasma, SELENOP is also expressed in tissues such as the testes, muscle, kidney, brain, small intestine, and colon (9, 10). Cells internalize extracellular, secreted SELENOP via receptor-mediated endocytosis, once SELENOP binds low-density lipoprotein receptor–related proteins (LRPs) on the cell surface (8, 11). LRP1 and LRP2 (also known as megalin) have been identified as the SELENOP receptors in muscle and kidney, respectively (12, 13), whereas LRP8 (also known as ApoER2) has been identified as the SELENOP receptor in bone, brain, and testes (14–16). However, the SELENOP receptor(s) in the colon and small intestine, where LRP1, LRP2, and LRP8 are lowly expressed, remains unknown (17).

In sporadic colorectal cancer (CRC), genetic and epigenetic alterations influenced by lifestyle, environmental, and dietary factors drive carcinogenesis through activation of oncogenes and inactivation of tumor suppressor genes (18). Conventional CRCs, which comprise 60%–85% of sporadic CRCs, are characterized by initial inactivation of the tumor suppressor gene adenomatous polyposis coli (*APC*) and resultant hyperactivation of WNT signaling (19). In canonical WNT signaling, a destruction complex targets cytoplasmic β-catenin for proteasomal degradation. Binding of WNT ligands to their coreceptors low-density lipoprotein receptor–related proteins 5 and 6 (LRP5/6) and frizzled (FZD) inhibits destruction complex activity and triggers nuclear translocation of β-catenin. In the nucleus, β-catenin binds T cell factor/lymphoid enhancer factor (TCF/LEF) transcription factors to induce transcription of WNT target genes (20). Importantly, upstream WNT ligands continue to activate WNT signaling, even in the context of downstream WNT signaling hyperactivation (e.g., *APC* loss of function) (21, 22).

In this study, we delineated tumor-promotive roles for SELENOP in sporadic CRC through amplification of canonical WNT signaling activity via specific interactions with LRP5/6. In human single-cell RNA-Seq (scRNA-Seq) data sets, we discovered progressive increases in *SELENOP* expression from stem to adenoma to carcinoma cells. To test our hypothesis that SELENOP promotes intestinal tumorigenesis, we defined the effects of *Selenop* KO in an *Apc*-dependent adenoma mouse model. Here, *Selenop* KO decreased colon tumor incidence and size. Additionally, *Selenop*-KO tumor organoids demonstrated reduced organoid formation and WNT target gene expression, which could be reversed by SELENOP overexpression. Moreover, SELENOP increased canonical WNT signaling activity in noncancer and colon cancer cell lines. In defining the mechanism, we identified a protein-protein interaction between SELENOP and LRP5/6 and mapped the specific LRP5/6 interaction domain on SELENOP. Furthermore, we established that SELENOP’s LRP5/6 interaction domain mediates its effects on canonical WNT signaling activity. Overall, our results position SELENOP as a modulator of canonical WNT signaling activity in sporadic CRC.

## Results

### SELENOP is predominantly expressed by differentiated epithelial cells in the normal colon and small intestine epithelium.

**Figure 1:**
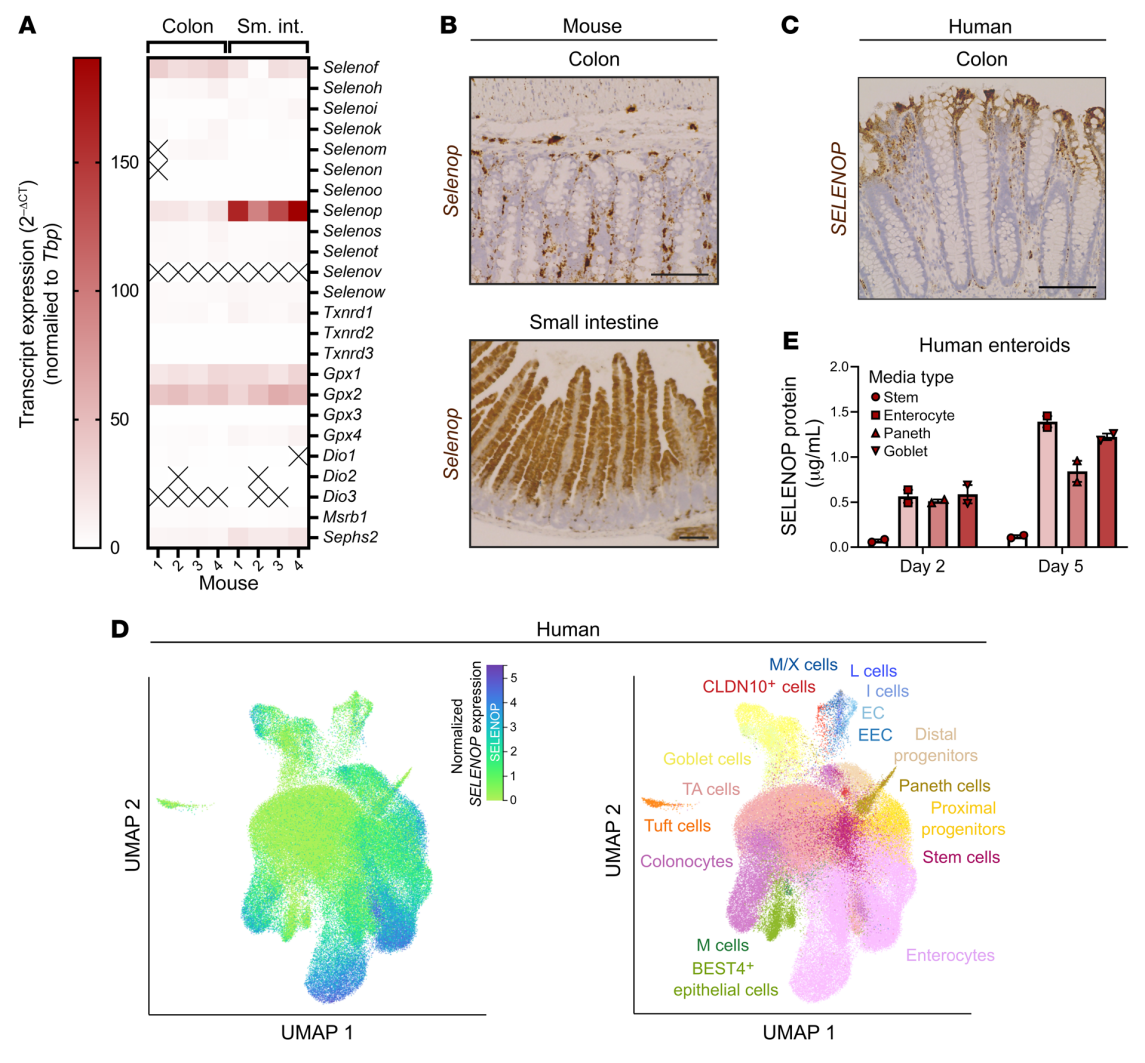
*SELENOP* is predominantly expressed by differentiated epithelial cells in the normal colon and small intestine epithelium. (**A**) RT-qPCR of mouse colon and small intestine (sm. int.) epithelial isolates for selenoproteins. *n* = 4 mice. (**B**) RNAscope of mouse colon and small intestine for *Selenop*. Representative images of colon (original magnification, ×20) and/or small intestine (original magnification, ×10). Scale bars: 100 μm. (**C**) RNAscope of human colon for *SELENOP*. Representative images (original magnification, ×20). Scale bar: 100 μm. (**D**) Gut Cell Atlas scRNA-Seq data from human colon and small intestine epithelium queried for *SELENOP*. EC, enterochromaffin; EEC, enteroendocrine; TA, transit-amplifying. *n* = 6 donors. (**E**) ELISA of conditioned media from human enteroids treated with the indicated media for SELENOP. Data were pooled from 2 independent experiments. Data are displayed as the mean ± SEM. EC, enterochromaffin cell; EEC, enteroendocrine cell; M; microfold; M/X, MLN^+^GHRL^+^; TA, transit-amplifying.

We first profiled the selenotranscriptome in WT mouse small intestine and colon epithelial isolates by reverse transcription quantitative PCR (RT-qPCR). *Selenop* was the most abundant selenoprotein mRNA in the small intestine epithelium (Figure 1A), in agreement with prior measurements of selenoprotein mRNA levels in whole small intestine tissue (23). *Selenop* was one of several highly expressed selenoprotein mRNAs, including selenoprotein F (*Selenof*), glutathione peroxidase 1 (*Gpx1*), and glutathione peroxidase 2 (*Gpx2*), in the small intestine and colon epithelium (Figure 1A). Additionally, we confirmed GPX1 (Supplemental Figure 1A) and GPX2 (Supplemental Figure 1B; supplemental material available online with this article; https://doi.org/10.1172/JCI165988DS1) protein expression in these tissues (for Supplemental Figure 1, A and B, see complete unedited blots in the supplemental material). We observed similar selenotranscript expression patterns in the Gut Cell Atlas scRNA-Seq data set (24) generated from normal human colon and small intestine epithelium (Supplemental Figure 2).

When we performed RNA ISH on WT mouse tissues with a validated *Selenop* RNAscope probe (Supplemental Figure 3), we predominantly detected *Selenop* in differentiated epithelial cells of the villi and crypts, as well as in stromal cells (Figure 1B). We observed a similar pattern of *SELENOP* expression in human colon tissues (Figure 1C). Together, these findings complement previously described *SELENOP* expression patterns in mouse and human colon tissues (25). In the Gut Cell Atlas scRNA-Seq data set (24), *SELENOP* was moderately to highly expressed throughout enterocyte and colonocyte populations, as well as in subsets of proximal progenitor, Paneth, goblet, and enteroendocrine cells (Figure 1D). To corroborate these observations, we subjected human small intestinal organoids (“enteroids”) to established directed differentiation protocols (26) and then measured SELENOP protein levels by ELISA. Indeed, we found that SELENOP protein was highly expressed among enteroids differentiated toward enterocytes, goblet cells, or Paneth cells (Figure 1E). We observed similar trends in *SELENOP* transcript expression in enteroids skewed toward the enterocyte, goblet cell, or Paneth cell lineages (Supplemental Figure 4).

### SELENOP expression progressively increases throughout conventional colorectal carcinogenesis.

**Figure 2:**
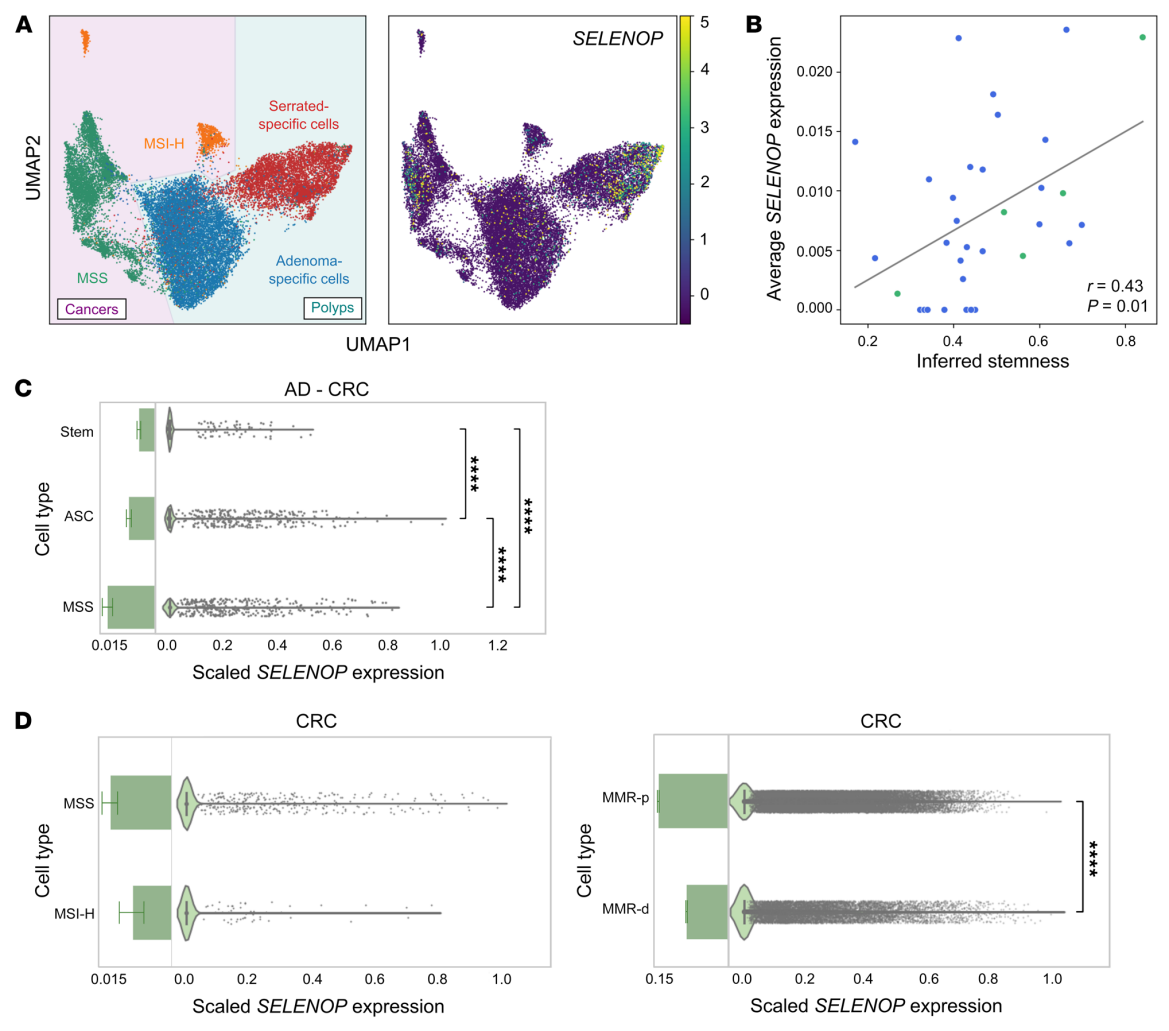
*SELENOP* expression progressively increases throughout conventional colorectal carcinogenesis. (**A** and **B**) scRNA-seq data from human colorectal polyps and cancers. (**A**) *SELENOP* expression in cell clusters. *n* = 62 polyps; *n* = 7 cancers; *n* = 149,116 cells. UMAP, uniform manifold approximation and projection. (**B**) *SELENOP* expression versus stemness inferred from CytoTRACE analysis. *n* = 29 polyps; *n* = 5 cancers. (**C**) scRNA-Seq data from human colorectal polyps or cancers and normal colon tissues (27). *SELENOP* expression by cell type. AD, adenoma. *n* = 34 normal samples; *n* = 29 polyps; *n* = 5 cancers. (**D**) scRNA-Seq data from human colorectal cancers (27, 30). *SELENOP* expression by tumor type. MMR-d, mismatch repair deficient; MMR-p, mismatch repair proficient. *n* = 2 MSI-H cancers; *n* = 5 MSS cancers (left); *n* = 32 MMR-d cancers; *n* = 28 MMR-p cancers (right). ****P < 0.0001, by Spearman’s rank correlation (**B**), Kruskal-Wallis test with a 2-sided Mann-Whitney *U* test (**C**), and 2-sided Mann-Whitney *U* test (**D**). Data are displayed as the mean ± SD.

We next evaluated *SELENOP* expression in colorectal polyps and cancers. For these analyses, we used a previously published scRNA-Seq data set of conventional adenomas (adenoma-specific cells [ASCs]), serrated polyps (serrated-specific cells [SSCs]), microsatellite stable (MSS) cancers, and microsatellite instability–high (MSI-H) cancers (27). Stem and absorptive cells are thought to represent the tumor-initiating cell types for conventional adenomas and serrated polyps, respectively, that can lead to MSS and MSI-H cancers (27). Here, we observed high *SELENOP* expression in subsets of ASCs, SSCs, and MSS cancer cells (Figure 2A). Moreover, in ASCs and MSS cancer cells, *SELENOP* expression was weakly correlated (*r* = 0.44, *P* = 0.01) with inferred stemness, as derived from Cellular Trajectory Reconstruction Analysis Using Gene Counts and Expression (CytoTRACE) analysis that computationally predicts cellular differentiation states from scRNA-Seq data (28) (Figure 2B).

When we integrated this data set with its corresponding patient-matched normal tissue data sets (Supplemental Figure 5A), we observed increases in *SELENOP* expression from normal crypt stem cells to ASCs to MSS cancer cells (Figure 2C). Similarly, in a single-nucleus RNA-Seq (snRNA-Seq) data set generated from patients with familial adenomatous polyposis (FAP) and from non-FAP patients (29) (Supplemental Figure 5B), *SELENOP* expression was greater in adenocarcinomas than in polyps or unaffected stem cells (Supplemental Figure 5C). We also noted higher *SELENOP* expression in SSCs than in absorptive cells; however, *SELENOP* expression did not differ between absorptive cells and MSI-H cancer cells (Supplemental Figure 5D). Although *SELENOP* expression levels did not differ (*P* = 0.263) between MSS and MSI-H cancers in this particular data set (27) (Figure 2A and Figure 2D), *SELENOP* expression was greater in mismatch repair–proficient (MMR-proficient) than MMR-deficient cancers in another scRNA-Seq data set (30) (Figure 2D), and this correlated with the proportion of stem-like cells present in each cancer type. Overall, these results suggest that upregulation of *SELENOP* expression throughout conventional colorectal carcinogenesis occurs as a function of stemness.

### Selenop KO decreases colon tumor incidence and size in Apc-dependent tumorigenesis.

**Figure 3:**
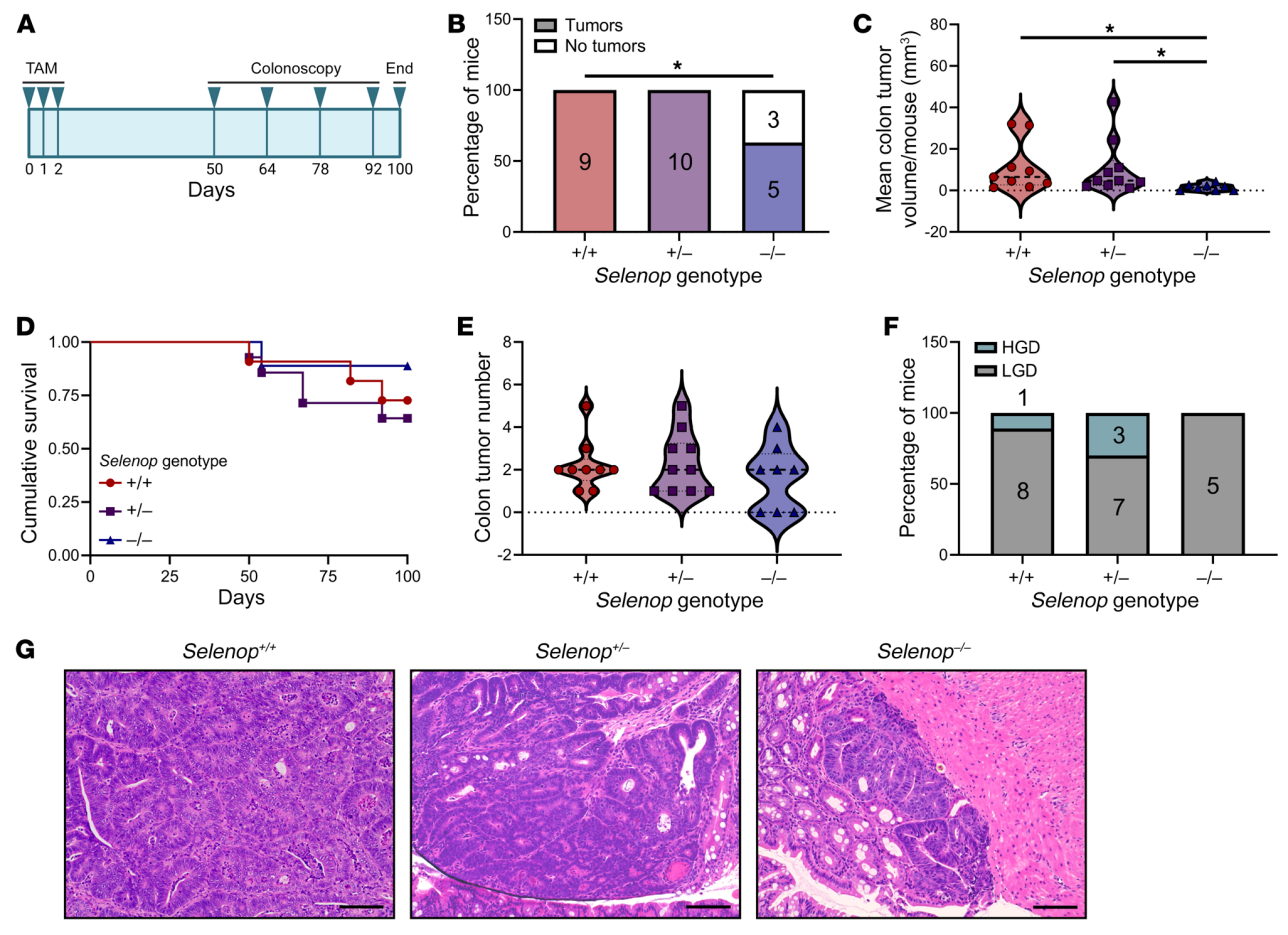
*Selenop* KO decreases colon tumor incidence and size in *Apc-*dependent tumorigenesis. (**A**) Schematic of murine tumorigenesis protocol. TAM, tamoxifen. (**B**) Colon tumor incidence, (**C**) colon tumor volume, (**D**) cumulative survival, (**E**) colon tumor numbers, (**F**) colon tumor dysplasia scores (HGD, high-grade dysplasia, LGD, low-grade dysplasia), and (**G**) histology of colon tumors from *Apc^ΔIE/+^ Selenop^+/+^* (*n* = 9), *Selenop^+/–^* (*n* = 10), and *Selenop^–/–^* (*n* = 8) mice. Original magnification, ×20. Scale bars: 100 μm. Data were pooled from 2 independent experiments. *P < 0.05, by Freeman-Halton test (**B** and **F**), Kruskal-Wallis test (**C** and **E**) with 2-sided Dunn’s multiple-comparison test (**C**), and log-rank test (**D**).

Since *SELENOP* upregulation correlated with the conventional adenoma-carcinoma sequence, we hypothesized that SELENOP deficiency would reduce stem cell–driven colorectal tumorigenesis. To model this, we crossed *Selenop^–/–^* mice (31) onto the *Lrig1-CreERT2/^+^ Apc^fl/+^* genetic background (32). Importantly, these mice were maintained on a defined, selenium-supplemented diet (1.0 mg selenium/kg) to control for micronutrient variations among different lots of standard chow (33) and avert the neurological dysfunction observed in *Selenop^–/–^* mice (34). The tamoxifen-inducible *Lrig1-CreERT2* driver facilitates the loss of 1 *Apc* allele in leucine-rich repeats and immunoglobulin-like domains 1–positive (*Lrig1*-positive) intestinal epithelial stem cells, and *Apc* loss of heterozygosity occurs in this model as in human CRC (35). Tamoxifen-induced *Lrig1-CreERT2/^+^ Apc^fl/+^ Selenop^+/+^*, *Selenop^+/–^*, and *Selenop^–/–^* cohorts (hereafter referred to as *Apc^ΔIE/+^ Selenop^+/+^*, *Selenop^+/–^*, and *Selenop^–/–^* mice) were monitored for tumor formation via colonoscopy and euthanized after 100 days (Figure 3A).

In the colon, we observed decreased tumor incidence (Figure 3B) and volume (Figure 3C) in *Apc^ΔIE/+^ Selenop^–/–^* mice as compared with *Apc^ΔIE/+^ Selenop^+/+^* or *Selenop^+/–^* mice, despite similar survival rates (Figure 3D), numbers (Figure 3E), and dysplasia severity (Figure 3, F and G). Similarly, in the small intestine, we observed decreased tumor areas (Supplemental Figure 6A) in *Apc^ΔIE/+^ Selenop^–/–^* mice as compared with *Apc^ΔIE/+^ Selenop^+/+^* or *Selenop^+/–^* mice, despite similar incidence rates (Supplemental Figure 6B), numbers (Supplemental Figure 6C), and dysplasia severity (Supplemental Figure 6, D and E). Altogether, these results propound tumor-promotive roles for SELENOP in *Apc*-dependent tumorigenesis.

### Selenop KO decreases tumoroid-forming capacity and WNT target gene expression.

**Figure 4:**
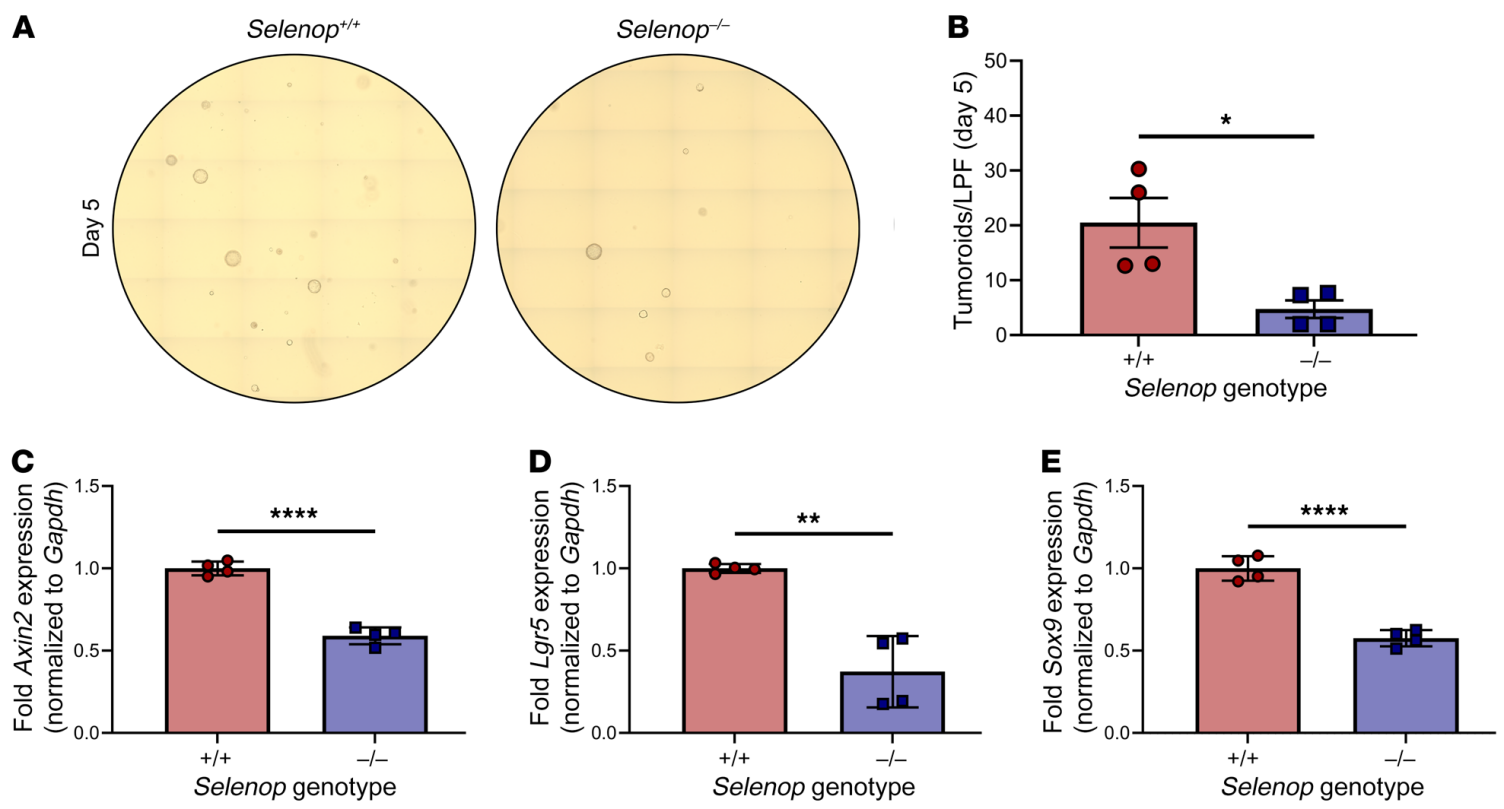
*Selenop* KO decreases tumoroid-forming capacity and WNT target gene expression. (**A** and **B**) *Apc^ΔIE/+^ Selenop^+/+^* or *Selenop^–/–^* tumoroids 5 days after enzymatic dissociation. (**A**) Representative ×10 tile scans. (**B**) Visible tumoroids per low-powered field (LPF). (**C**–**E**) RT-qPCR for (**C**) *Axin2*, (**D**) *Lgr5*, and (**E**) *Sox9* of *Apc^ΔIE/+^ Selenop^+/+^* or *Selenop^–/–^* tumoroids. Data were pooled from 2 independent experiments with 2 mice per genotype. *P < 0.05, **P < 0.01, and ****P < 0.0001, by 2-sided, unpaired *t* test. Data are displayed as the mean ± SEM.

To interrogate these phenotypes further, we established tumor organoids (“tumoroids”) from *Apc^ΔIE/+^ Selenop^+/+^* and *Selenop^–/–^* adenomas. Since *Apc^ΔIE/+^ Selenop^–/–^* mice developed smaller colon tumors than did *Apc^ΔIE/+^ Selenop^+/+^* mice in vivo, we hypothesized that *Apc^ΔIE/+^ Selenop^–/–^* tumoroids would exhibit defects in organoid formation ex vivo. To test this, we dissociated *Apc^ΔIE/+^ Selenop^+/+^* and *Selenop^–/–^* tumoroids, plated equivalent cell numbers, imaged them after 5 days (Figure 4A), and quantified the viable tumoroids (Figure 4B). Indeed, *Apc^ΔIE/+^ Selenop^–/–^* tumoroids showed lower single-cell plating efficiency than did *Apc^ΔIE/+^ Selenop^+/+^* tumoroids (Figure 4B).

As untransformed intestinal crypts require exogenous WNT stimulation to form organoids ex vivo (36), we hypothesized that *Apc^ΔIE/+^ Selenop^–/–^* tumoroids would exhibit lower WNT activity than *Apc^ΔIE/+^ Selenop^+/+^* tumoroids. In fact, *Apc^ΔIE/+^ Selenop^–/–^* tumoroids had lower levels of the WNT target genes *Axin2*, leucine-rich repeat-containing GPCR (*Lgr5*), and sex-determining region Y-box transcription factor 9 (*Sox9*) than *Selenop^+/+^* tumoroids (Figure 4, C–E). Thus, *Apc^ΔIE/+^ Selenop^–/–^* tumoroids recapitulated aspects of the tumor phenotypes observed in *Apc^ΔIE/+^ Selenop^–/–^* mice.

### SELENOP restoration increases tumoroid-forming capacity and WNT target gene expression.

**Figure 5:**
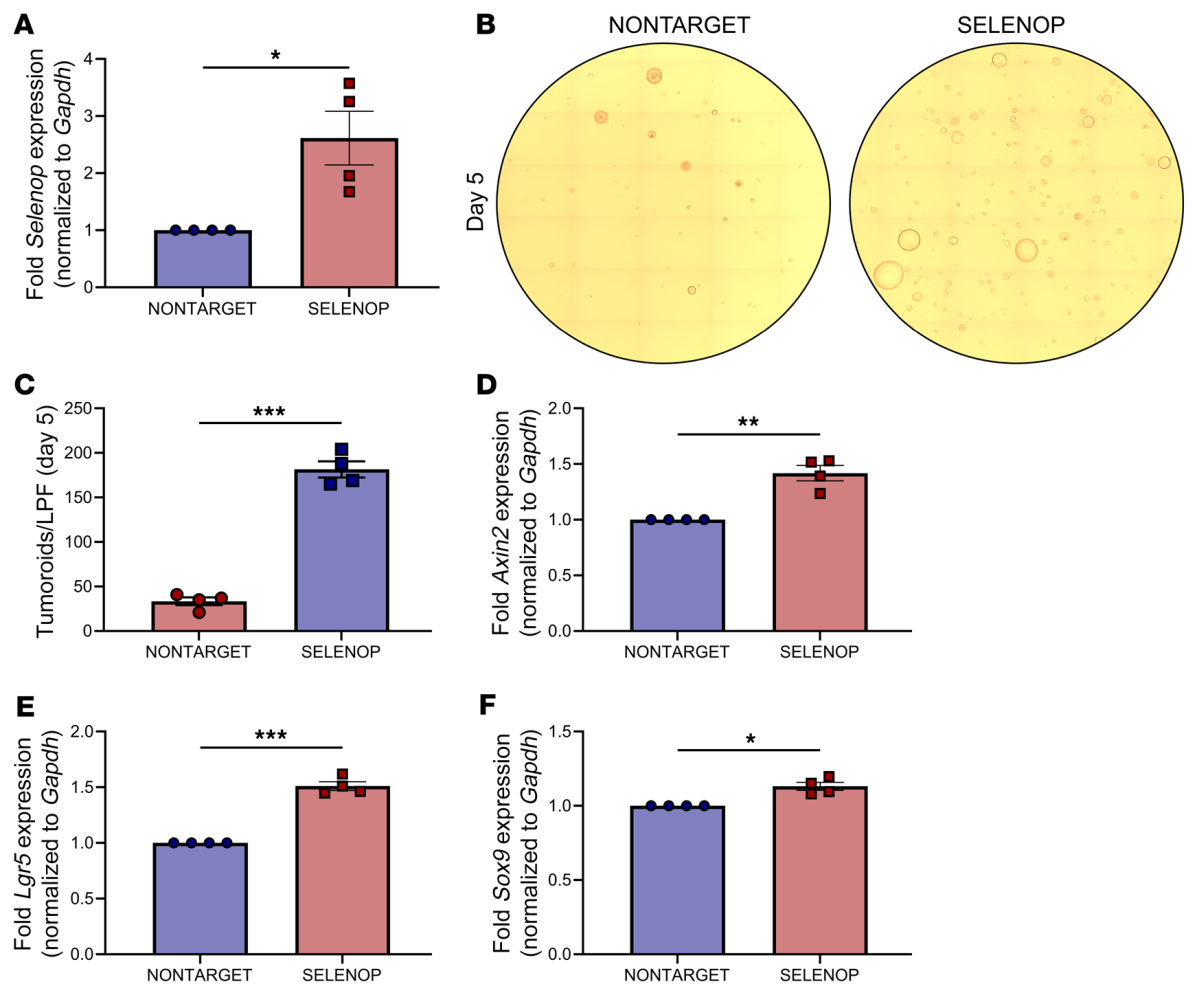
SELENOP restoration increases tumoroid-forming capacity and WNT target gene expression. (**A**) RT-qPCR for *Selenop* of *Apc^ΔIE/+^ Selenop^+/+^*-dCas9-VP64-NONTARGET or SELENOP tumoroids. (**B** and **C**) *Apc^ΔIE/+^ Selenop^+/+^*-dCas9-VP64-NONTARGET or SELENOP tumoroids 5 days after enzymatic dissociation. (**B**) Representative ×10 tile scans. (**C**) Visible tumoroids per LPF. (**D**–**F**) RT-qPCR for (**D**) *Axin2*, (**E**) *Lgr5*, and (**F**) *Sox9* in *Apc^ΔIE/+^ Selenop^+/+^*-dCas9-VP64-NONTARGET or SELENOP tumoroids. Data were pooled from 4 independent experiments. *P < 0.05, **P < 0.01, and ***P < 0.001, by 2-sided, paired *t* test. Data are displayed as the mean ± SEM.

As *Selenop* deficiency dampened WNT tone in tumoroids, we hypothesized that SELENOP restoration would reverse this phenotype. To investigate this, we transduced *Apc^ΔIE/+^ Selenop^+/+^* tumoroids, in which *Selenop* expression was substantially downregulated (Supplemental Figure 7), with a nuclease-deficient Cas9 (dCas9) fused to a transcriptional activator (VP64) and nontarget or *Selenop* promoter–targeted sgRNAs, to drive *Selenop* transcription from the endogenous locus (Figure 5A). When we dissociated and plated *Apc^ΔIE/+^ Selenop^+/+^*-dCas9-VP64-NONTARGET and SELENOP tumoroids as single cells, more SELENOP-overexpressing cells formed tumoroids after 5 days, as compared with control cells (Figure 5, B and C). As we and others have reported that additional WNT stimulation increased tumoroid growth even after *Apc* loss of function (21, 22), we also measured levels of WNT target transcripts by RT-qPCR. Here, SELENOP-overexpressing tumoroids displayed higher *Axin2*, *Lgr5*, and *Sox9* transcript levels than did control tumoroids (Figure 5, D–F). Altogether, these results demonstrate that SELENOP overexpression rescued the effects of *Selenop* deficiency on tumoroid-forming capacity and WNT target gene expression.

### SELENOP increases WNT target gene expression in human tumoroids.

Additionally, we tested the effects of SELENOP treatment on WNT target gene expression in human tumoroid lines established from patients with stage II/III CRC (Supplemental Table 6). Although WNT target transcript levels differed among tumoroid lines, treatment with purified human SELENOP increased *SOX9* levels in lines 32385, 35349, and 40299; *LGR5* levels in line 35349; and *AXIN2* levels in line 40299 (Supplemental Figure 8). Thus, SELENOP also amplified WNT signaling activity in human CRC tumoroids.

### SELENOP increases canonical WNT signaling activity in noncancer and colon cancer cell lines.

**Figure 6:**
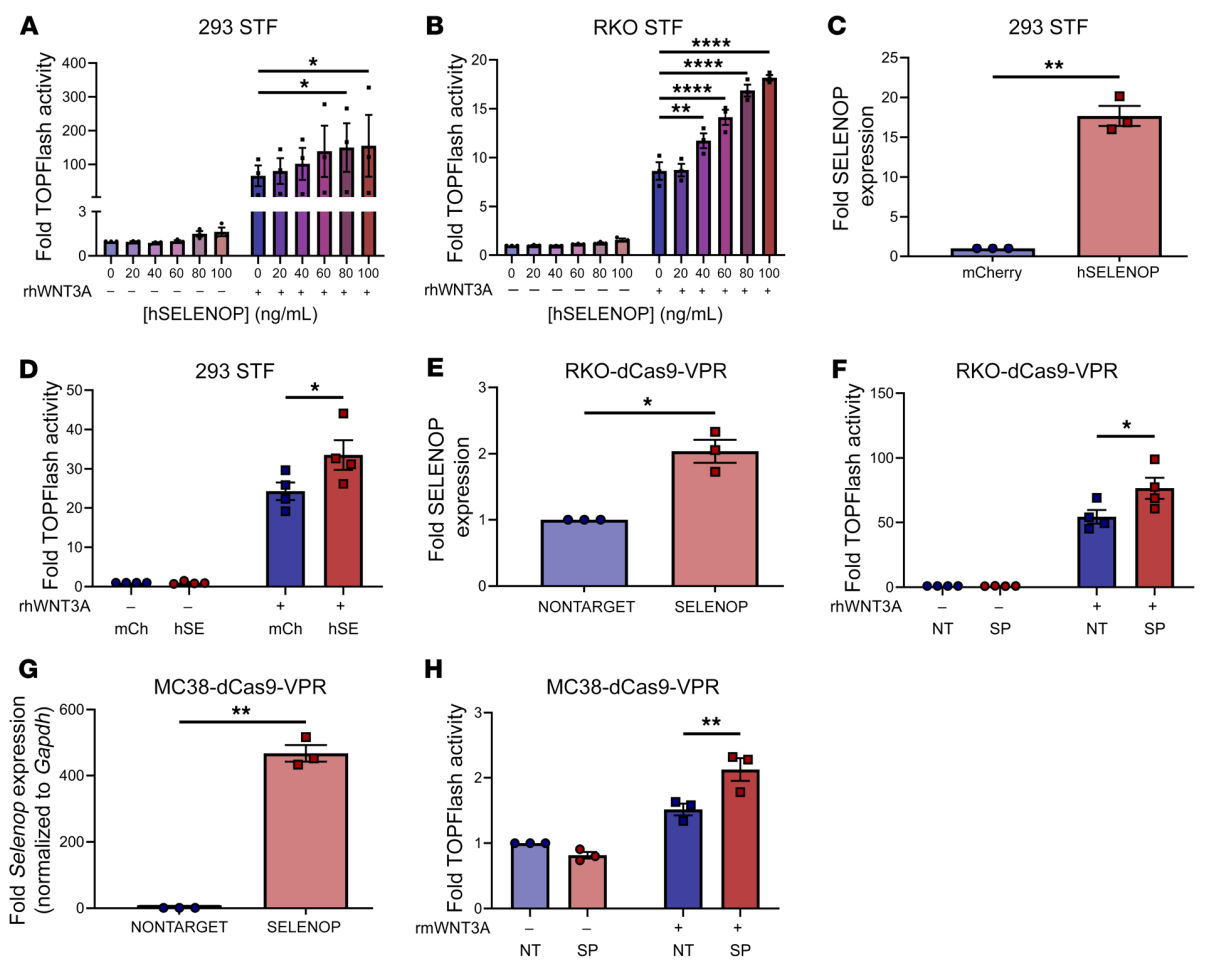
SELENOP increases canonical WNT signaling activity in noncancer and colon cancer cell lines. (**A** and **B**) TOPFlash activity of (**A**) 293 STF and (**B**) RKO STF cells treated or not with rhWNT3A and the indicated concentrations of hSELENOP. (**C**) ELISA for SELENOP of 293 STF-mCherry or hSELENOP conditioned media. (**D**) TOPFlash activity of 293 STF-mCherry or hSELENOP cells treated or not with rhWNT3A. hSE, hSELENOP; mCh, mCherry. (**E**) ELISA for SELENOP of RKO-dCas9-VPR-NONTARGET or SELENOP conditioned media. (**F**) TOPFlash activity of RKO-dCas9-VPR-NONTARGET (NT) or SELENOP (SP) cells treated or not with rhWNT3A. (**G**) RT-qPCR for *Selenop* of MC38-dCas9-VPR-NONTARGET or SELENOP cells. (**H**) TOPFlash activity of MC38-dCas9-VPR-NONTARGET or SELENOP cells treated or not with rmWNT3A. Data were pooled from 3–4 independent experiments. *P < 0.05, **P < 0.01, and ****P < 0.0001, by 2-way, repeated-measures ANOVA with 2-sided Dunnett’s multiple-comparison test (**A** and **B**), 2-sided, paired *t* test (**C**, **E**, and **G**), and 2-way, repeated-measures ANOVA with 2-sided Šidák’s multiple-comparison test (**D**, **F**, and **H**). Data are displayed as the mean ± SEM.

As SELENOP under- and overexpression in tumoroids decreased and increased WNT target gene expression, respectively, we hypothesized that SELENOP might directly amplify WNT signaling activity. To investigate this, we used 293 Super TOPFlash (STF) cells, which stably express a luciferase reporter of β-catenin/TCF/LEF-mediated transcription that serves as a direct readout of canonical WNT signaling activity (37). In 293 STF cells, combinatorial treatment with SELENOP and WNT3A increased TOPFlash activity to a greater extent than did treatment with WNT3A alone (Figure 6A). As 293 STF cells are a noncancer cell line, we subsequently generated RKO (human colon adenocarcinoma) STF cells to confirm this observation and contextualize these findings in CRC. Importantly, RKO cells possess both WT APC and β-catenin and, as such, display intact WNT signaling (38). Similarly, exogenous SELENOP amplified WNT3A-induced TOPFlash activity in RKO STF cells (Figure 6B).

As SELENOP is a secreted protein, we hypothesized that secreted SELENOP would increase WNT signaling by an autocrine and/or paracrine mechanism. Indeed, lentiviral SELENOP overexpression in 293 STF cells (Figure 6C) promoted WNT3A-induced TOPFlash activity (Figure 6D). Similarly, CRISPR activation–mediated (CRISPRa-mediated) SELENOP overexpression in RKO cells (Figure 6E) or MC38 (mouse colon adenocarcinoma) cells (Figure 6G) augmented WNT3A-induced TOPFlash activity (Figure 6F, Figure 6H). Overall, it appears that exogenous or endogenous SELENOP augmented canonical WNT signaling.

### SELENOP interacts with LRP6.

**Figure 7:**
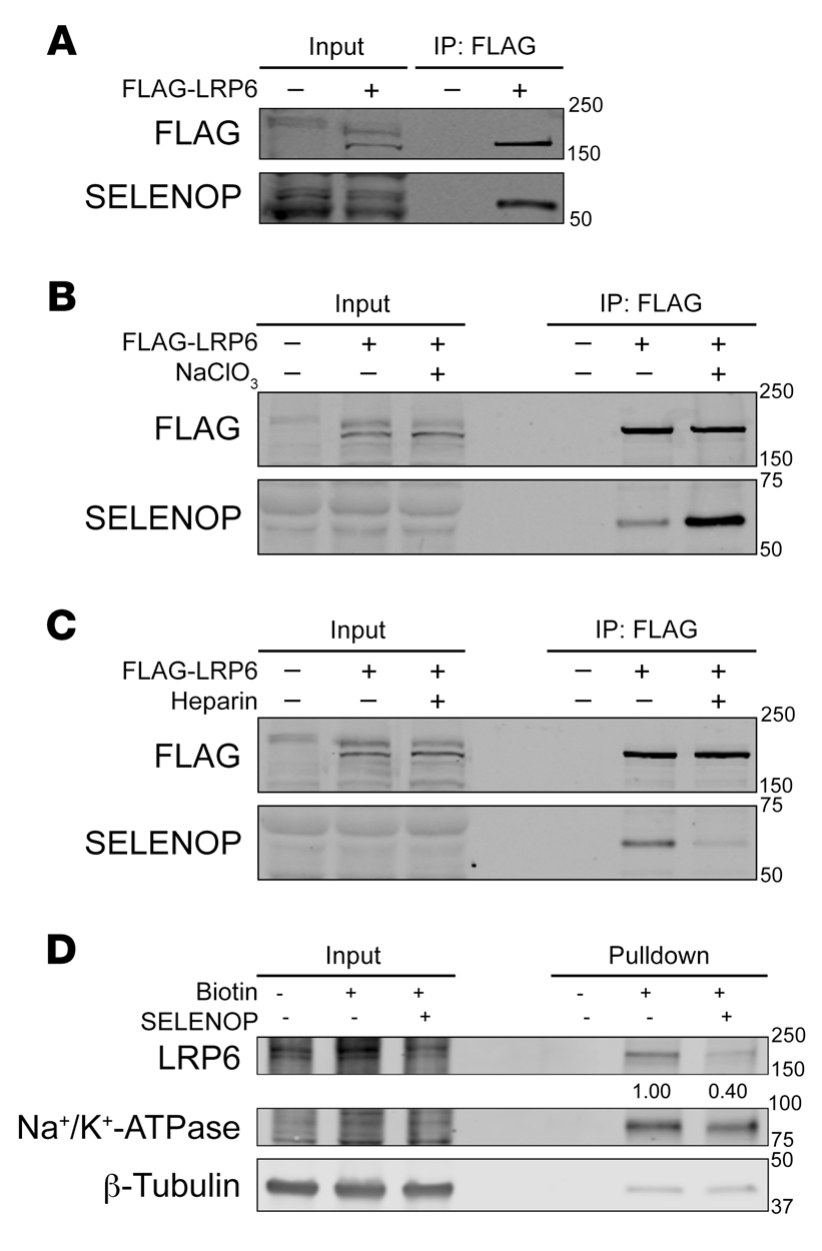
SELENOP interacts with LRP6. (**A**) Western blot for FLAG and SELENOP of FLAG IPs from 293T or 293T-FLAG-LRP6 cells. (**B**) Western blot for FLAG and SELENOP of FLAG IPs from 293T or 293T-FLAG-LRP6 cells treated or not with sodium chlorate (NaClO_3_). (**C**) Western blot for FLAG and SELENOP of FLAG IPs from 293T or 293T-FLAG-LRP6 cells treated or not with heparin. (**D**) Western blot for LRP6, Na^+^/K^+^-ATPase (plasma membrane loading control), and β-tubulin (whole-cell loading control) of cell-surface biotinylation and isolation from 293T cells treated or not with SELENOP-conditioned media. Data are representative of 3 independent experiments.

We next interrogated the mechanism by which SELENOP increased canonical WNT signaling. Interestingly, exogenous SELENOP increased TOPFlash activity even after *APC* knockdown in 293 STF cells (Supplemental Figure 9; see complete unedited blots in the supplemental material). As WNTs bind LRP5/6 and FZD coreceptors to activate WNT signaling (39), while SELENOP binds tissue-specific LRP1, LRP2, or LRP8 receptors for receptor-mediated endocytosis (12, 15, 16, 40, 41), we hypothesized that SELENOP modifies WNT signaling through interactions with LRP5/6. To test this hypothesis, we used 293T cells that stably expressed FLAG-tagged endogenous LRP6, and we observed that SELENOP co-immunoprecipitated with FLAG-LRP6 in these cells (Figure 7A; see complete unedited blots in the supplemental material). We confirmed the SELENOP-LRP6 interaction by proximity ligation assay in 293T cells transfected with FLAG-tagged mouse LRP6 (FLAG-mLRP6) and V5-tagged mouse SELENOP (V5-mSELENOP) overexpression constructs (Supplemental Figure 10).

As SELENOP is widely thought to bind heparan sulfate proteoglycans (HSPGs) (42), and HSPGs deliver WNT modulators and ligands to LRP5/6 (43), we hypothesized that HSPGs facilitate SELENOP-LRP6 interactions. Surprisingly, inhibition of HSPG synthesis (via sodium chlorate [NaClO_3_] treatment) markedly enhanced co-IP of SELENOP and FLAG-LRP6 in 293T-FLAG-LRP6 cells (Figure 7B; see complete unedited blots in the supplemental material). Conversely, treatment with heparin prevented SELENOP and FLAG-LRP6 co-IP in these cells (Figure 7C; see complete unedited blots in the supplemental material). Furthermore, we investigated whether SELENOP accelerates LRP5/6 recycling to potentiate WNT signaling. We tested this hypothesis through biotinylation and isolation of cell-surface proteins with and without SELENOP treatment. Indeed, we found that SELENOP decreased cell-surface LRP6 levels (Figure 7D; see complete unedited blots in the supplemental material). Thus, SELENOP interacted with LRP6 (unless sequestered by HSPGs), promoted LRP6 internalization, and thus amplified WNT signaling.

### SELENOP^U258-U299^ mediates SELENOP-LRP5/6 interactions and SELENOP-induced WNT signaling augmentation.

**Figure 8:**
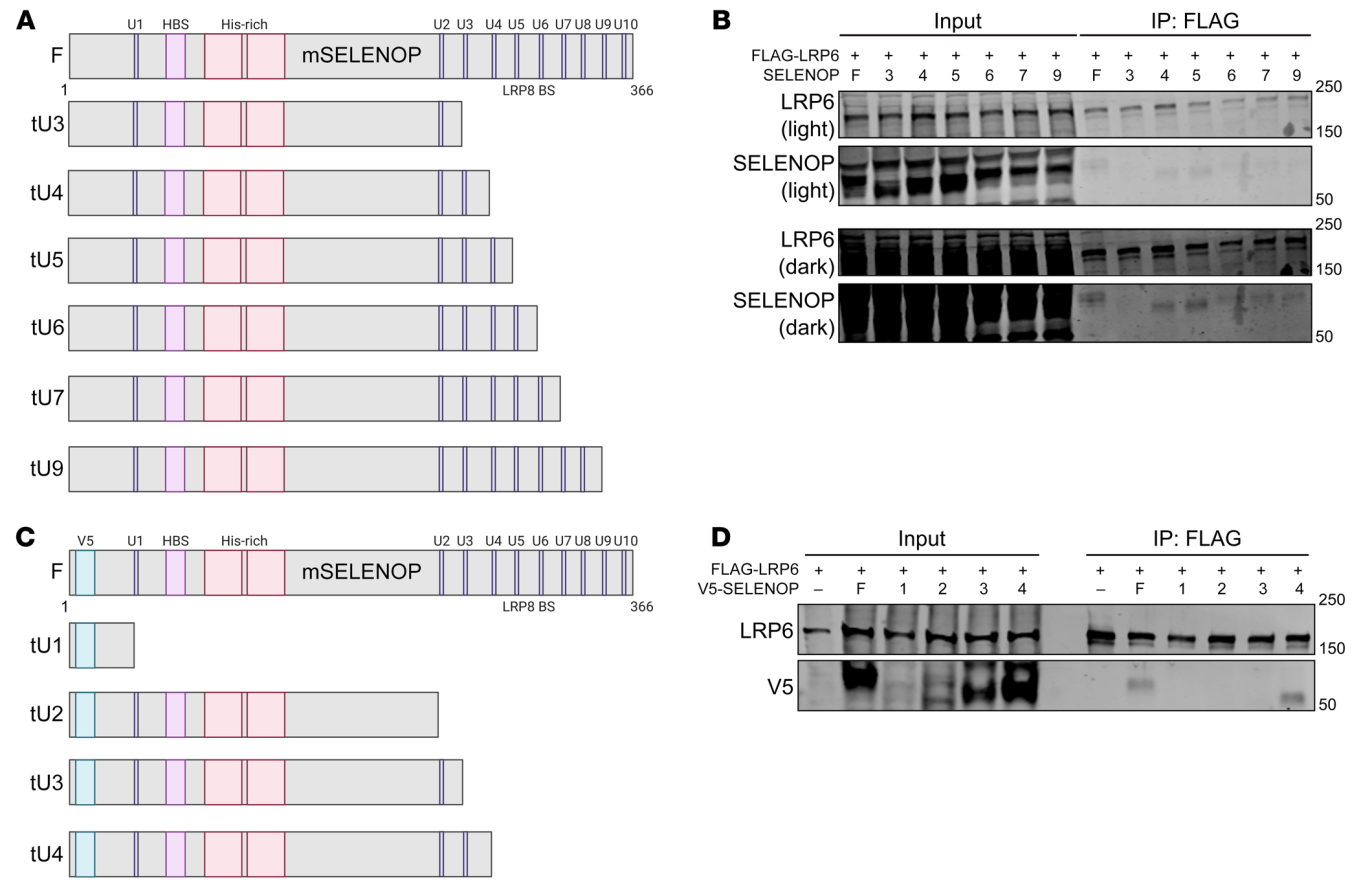
Longer SELENOP isoforms interact with LRP6. (**A**) Schematic of mouse SELENOP truncation (t) constructs. (**B**) Western blot for LRP6 and SELENOP of FLAG IPs from 293T cells cotransfected with FLAG-mLRP6 and full-length or truncated (at selenocysteine [U] number) mSELENOP. (**C**) Schematic of V5-tagged mouse SELENOP truncation constructs. (**D**) Western blot for LRP6 and V5 of FLAG IPs from 293T cells cotransfected with FLAG-mLRP6 and full-length or truncated (at U number) V5-mSELENOP. Data are representative of 3 independent experiments. F, full-length; HBS, heparin-binding site; His-rich, histidine-rich region; LRP8 BS, LRP8-binding site.

We next mapped the SELENOP-LRP6 interaction on SELENOP using FLAG-mLRP6 and mSELENOP overexpression constructs truncated (t) at SELENOP’s third, fourth, fifth, sixth, seventh, or ninth selenocysteine (U) (Figure 8A). As expected, full-length mSELENOP co-immunoprecipitated with FLAG-mLRP6 in 293T cells. Interestingly, only truncation at SELENOP’s third selenocysteine uncoupled the SELENOP-LRP6 interaction (Figure 8B; see complete unedited blots in the supplemental material). To further refine the LRP6 interaction domain on SELENOP, we generated V5-mSELENOP overexpression constructs truncated (t) at SELENOP’s first, second, third, or fourth selenocysteine (U) (Figure 8C). Both full-length and tU4 V5-mSELENOP co-immunoprecipitated with FLAG-mLRP6 in 293T cells; however, truncation at SELENOP’s first, second, or third selenocysteine uncoupled this interaction (Figure 8D; see complete unedited blots in the supplemental material).

**Figure 9:**
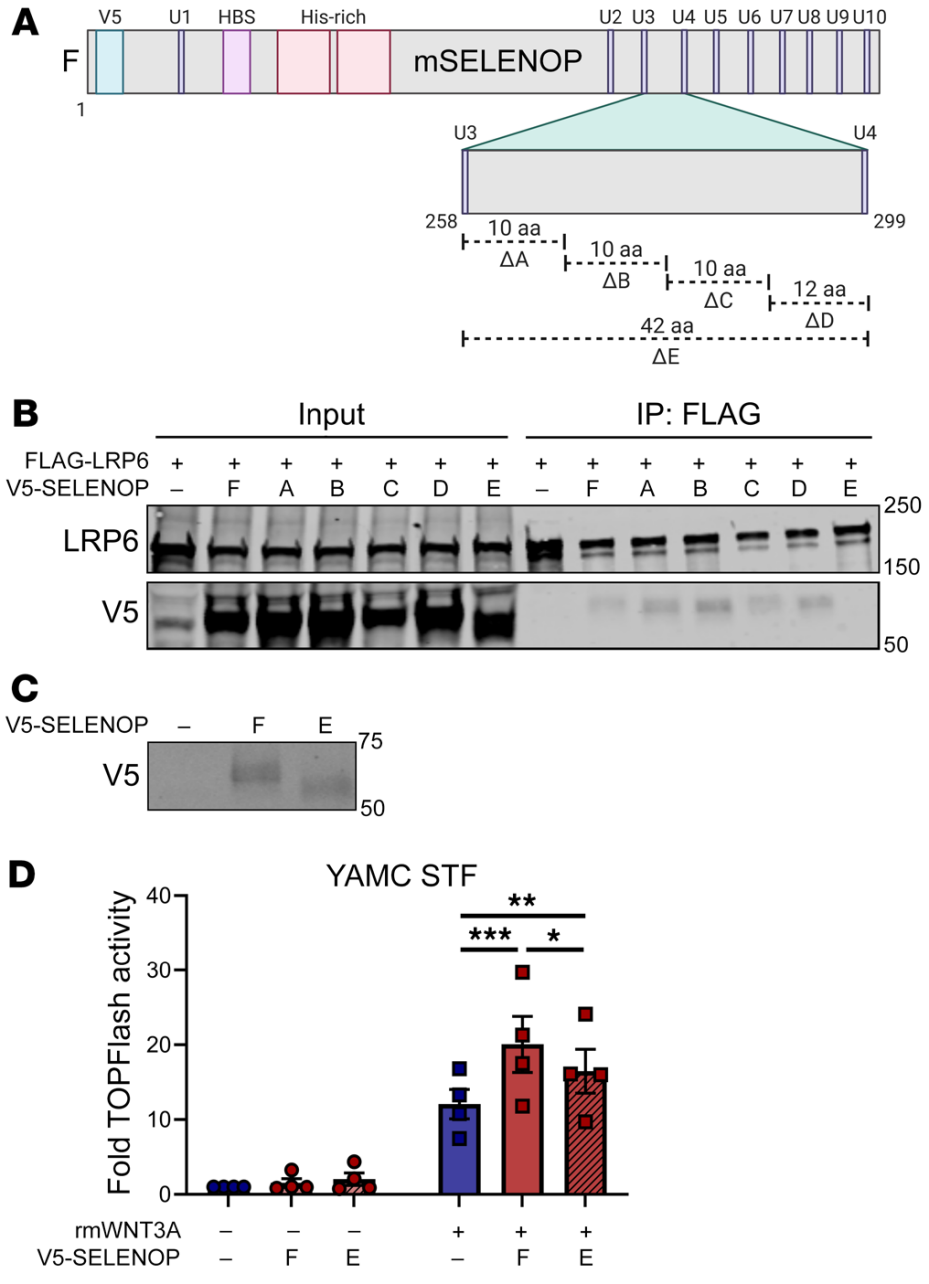
SELENOP^U258–U299^ mediates the SELENOP-LRP6 interaction and SELENOP-induced WNT signaling augmentation. (**A**) Schematic of V5-tagged mouse SELENOP deletion constructs. ΔA, Δ258-267; ΔB, Δ268-277; ΔC, Δ278-287; ΔD, Δ288-299; ΔE, Δ258-299. (**B**) Western blot for LRP6 and V5 of FLAG IPs from 293T cells cotransfected with FLAG-mLRP6 and full-length or mutant (A–E) V5-mSELENOP. (**C**) Western blot for V5 and (**D**) TOPFlash activity of YAMC STF cells transduced with full-length or LRP5/6-uncoupling (E) V5-mSELENOP. Representative (**B** and **C**) or pooled (**D**) data from 3–4 independent experiments. *P < 0.05, **P < 0.01, and ***P < 0.001, by 2-way, repeated-measures ANOVA with 2-sided Tukey’s multiple-comparison test. Data are displayed as the mean ± SEM.

We next generated V5-mSELENOP overexpression constructs with sequential, approximately 10 aa deletions (Δ) between SELENOP’s third (U258) and fourth (U299) selenocysteines, or 42 aa deletions (Δ) from U258 to U299 (Figure 9A). Interestingly, full-length, Δ258-267, Δ268-277, Δ278-287, and Δ288-299 V5-mSELENOP all co-immunoprecipitated with FLAG-mLRP6. Only deletion of the entire region from U258 to U299 uncoupled the SELENOP-LRP6 interaction (Figure 9B; see complete unedited blots in the supplemental material). As LRP6 and LRP5 share approximately 70% sequence identity (44), we hypothesized that SELENOP interacts with LRP5 through its U258-U299 domain. Indeed, we found that full-length, but not Δ258-299 V5-mSELENOP, co-immunoprecipitated with FLAG-mLRP5 (Supplemental Figure 11; see complete unedited blots in the supplemental material).

To test our hypothesis that SELENOP increases canonical WNT signaling activity through these specific LRP5/6 interactions, we performed TOPFlash assays on YAMC (immortalized mouse colon) STF cells transduced with full-length or LRP5/6-uncoupling (Δ258-299) V5-mSELENOP overexpression constructs (Figure 9C; see complete unedited blots in the supplemental material). As expected, overexpression of full-length V5-mSELENOP increased WNT3A-induced TOPFlash activity; however, overexpression of LRP5/6-uncoupling V5-mSELENOP decreased this effect (Figure 9D). Altogether, these results indicate that SELENOP^U258-U299^ mediates SELENOP-LRP5/6 interactions to promote WNT signaling activity.

## Discussion

In this study, we defined the role of SELENOP in sporadic colorectal carcinogenesis, which is predominantly initiated by mutations that hyperactivate the WNT signaling pathway. We observed increases in *SELENOP* expression throughout conventional adenoma to carcinoma progression. To test the functional consequences of *Selenop* deficiency on intestinal tumorigenesis, we used a mouse model in which intestinal epithelium–specific deletion of the tumor suppressor *Apc* and concomitant WNT signaling hyperactivation drive adenoma formation. In this model, *Selenop* KO was tumor protective. Underlying these phenotypes, we discovered a mechanism in which SELENOP modulated canonical WNT signaling activity through specific interactions with the WNT coreceptors LRP5/6.

We identified *Selenop* as the most highly expressed selenotranscript in the normal mouse small intestine epithelium, consistent with a selenotranscriptomic profile of whole mouse small intestine (23). To the best of our knowledge, we are the first to characterize selenoprotein mRNA expression specifically in the mouse colon and small intestine epithelium. When we examined *SELENOP* localization in situ, we observed a gradient of epithelial *SELENOP* expression up the crypt axis, as well as stromal *SELENOP* expression, in both mouse and human tissues. This expression pattern confirms prior findings in rat, mouse, and human small intestine/colon tissues and supports *SELENOP*’s recently proposed role as a crypt axis marker (9, 10, 25).

Our analyses revealed increases in *SELENOP* expression from tumor-initiating stem cells to adenomatous polyps and MSS cancers. Although others have reported reductions in *SELENOP* expression in colorectal tumors as compared with normal colon tissues (45–48), these studies did not stratify *SELENOP* expression by epithelial cell type and thus failed to account for the *SELENOP* expression gradient from crypt base to top in the normal colon. Namely, in comparisons with bulk normal colon tissues, we believe strong *SELENOP* expression in stromal and differentiated epithelial cells obscures the detection of meaningful, albeit subtle, differences in *SELENOP* expression from tumor-initiating cells to polyps and cancers.

While *SELENOP* expression was still lower in MSS cancers than in differentiated epithelial cells, we hypothesize that *SELENOP* upregulation throughout progression to malignancy fortifies tumor-promotive WNT signaling activity. Unlike in conventional CRCs, *SELENOP* expression was increased in serrated polyps, but not MSI-H cancers, as compared with tumor-initiating absorptive cells. Moreover, MMR-deficient tumors demonstrated decreased *SELENOP* expression as compared with MMR-proficient tumors. While beyond the scope of the current study, these intriguing results raise the possibility that SELENOP plays distinct roles in conventional versus serrated colorectal carcinogenesis.

In an *Apc*-dependent mouse adenoma model, *Selenop* KO reduced colon tumor size and incidence. Although SELENOP remains relatively understudied in sporadic CRC, the literature supports distinct roles for different selenoproteins in azoxymethane-induced (AOM-induced) experimental CRC. For example, transgenic mice with a mutation in the selenocysteine transfer RNA (tRNA) gene that inhibits selenocysteine synthesis, and thus reduces global selenoprotein production, developed fewer early neoplastic lesions called aberrant crypt foci (ACF) than did WT mice after AOM treatment (49). Similarly, *Gpx2*- or *Selenof*-KO mice developed fewer ACFs than WT mice after AOM treatment; in the case of *Gpx2-*KO mice, this corresponded with a decrease in tumor numbers (50, 51). In contrast, *Selenop-*KO mice developed more ACFs than did *Selenop*-WT mice after AOM treatment, although ACF progression to adenomas was not reported in this study (10). Importantly, studies that use ACFs as a primary readout of experimental tumorigenesis warrant cautious interpretation, as ACFs, while widely considered CRC precursors, have been demonstrated to regress spontaneously in several animal models (52–54). To the best of our knowledge, we are the first to investigate the effects of *Selenop* KO on adenoma, not ACF, development in a genetically, not chemically, induced CRC mouse model.

As in sporadic CRC models, current evidence suggests that different selenoproteins modify colitis-associated carcinoma (CAC) by distinct mechanisms. In the AOM/dextran sodium sulfate (DSS) experimental CAC model, *Gpx2-* or *Gpx3-*KO mice developed more tumors than did WT mice (55, 56). In contrast, *Selenof-*KO mice developed similar numbers of tumors, yet fewer ACFs, as compared with WT mice after AOM/DSS treatment (57). Notably, *Selenop-*KO mice developed fewer, smaller tumors than did *Selenop*-WT mice after an AOM/DSS protocol (10), which partially parallels our findings in experimental CRC. Additionally, *Selenop-*KO tumors from this CAC model displayed dysregulated WNT signaling, including transcriptional upregulation of the known WNT antagonists secreted frizzled-related proteins (SFRPs) 4 and 5 (10). Similarly, our *Apc^ΔIE/+^ Selenop^–/–^* tumoroids showed defects in organoid formation and decreases in WNT target gene expression that could be reversed by SELENOP restoration. Thus, SELENOP may play similar roles in CAC and sporadic CRC.

We discovered that SELENOP is a modulator of canonical WNT signaling activity through interactions with the WNT coreceptors LRP5/6. Although SELENOP’s effects on WNT signaling activity were previously undescribed, the literature supports roles for selenium itself as both a positive and negative regulator of WNT signaling activity. For example, both sodium selenate and selenomethionine administration activated WNT signaling in hippocampus tissue and primary neurons from a mouse model of Alzheimer’s disease (58, 59). However, selenomethionine treatment inhibited WNT signaling in HT-29 human colorectal adenocarcinoma cells (60). Similarly, selenium deficiency upregulated the transcription of WNT pathway targets and components in the normal mouse colon (61). Thus, the effects of selenium on WNT signaling activity may depend on tissue and disease context.

LRP1, LRP2, and LRP8 mediate SELENOP uptake in different tissues (12–16). Among these known SELENOP receptors, the interactions between SELENOP and LRP8 are well studied. SELENOP’s LRP8 interaction domain was previously mapped to 3 specific residues (Cys^343^, Gln^344^, Cys^345^) within the region between SELENOP’s fifth and sixth selenocysteines (62). As we mapped SELENOP’s LRP5/6 interaction domain to the 42 aa between SELENOP’s third and fourth selenocysteines (Sec^258^-Sec^299^), SELENOP binds LRP8 and LRP5/6 with distinct sites. In addition to LRP binding sites, SELENOP contains one well-defined (Leu^79^-Leu^84^) and 2 putative, histidine-rich (Thr^178^-Lys^189^ and His^194^-Gln^234^) heparin binding sites (42). As such, SELENOP is widely thought to bind cell-surface HSPGs (11). However, pretreatment with heparin failed to disrupt LRP8-SELENOP interactions (62). In contrast, pretreatment with heparin prevented LRP6-SELENOP interactions, and inhibition of HSPG synthesis promoted LRP6-SELENOP interactions. Thus, HSPGs may sequester SELENOP from LRP5/6, as they do other WNT modulators and ligands to fine-tune WNT signaling activity (43).

Although the SELENOP receptor(s) in the gastrointestinal tract remain unidentified, *LRP5* and *LRP6* are expressed at much higher levels than *LRP1*, *LRP2*, or *LRP8* in the small intestine and colon (24, 63). Therefore, LRP5/6 may represent bona fide receptors for SELENOP uptake in the gut. Our finding that SELENOP decreased cell-surface LRP6 levels raises the intriguing possibility that LRP6 mediates SELENOP internalization directly. As SELENOP’s expression pattern opposes the WNT3A gradient along the crypt/villus axis, perhaps LRP6 shuttles SELENOP into WNT^hi^, SELENOP^lo^ crypt base cells to facilitate synthesis of other selenoproteins and further amplify WNT signaling activity.

Taken together, our results present a role for SELENOP in WNT signaling modulation in the intestine, and perhaps in other tissues as well. Thus, our findings add yet another layer of complexity to the multimodal mechanisms of WNT signaling regulation in the intestine. This justifies further research into SELENOP’s contributions to sporadic colorectal carcinogenesis.

## Methods

Additional details can be found in the Supplemental Methods.

### RNA isolation, cDNA synthesis, and RT-qPCR.

Colon and small intestine epithelia were isolated as previously described (64). Cells and organoids were homogenized in TRIzol Reagent (15596018, Invitrogen, Thermo Fisher Scientific) prior to RNA isolation with the RNeasy Mini (74106, QIAGEN) or Micro (74004, QIAGEN) Kit, as appropriate. cDNA was synthesized from 2 μg total RNA with qScript cDNA SuperMix (95048100, Quantabio). TaqMan RT-qPCR was performed in triplicate with the TaqMan probes listed in Supplemental Table 1 (Applied Biosystems) and TaqMan Universal PCR Master Mix (4304437, Applied Biosystems). SYBR Green RT-qPCR was performed in triplicate using the primers listed in Supplemental Table 2 (Integrated DNA Technologies) and PerfeCTa SYBR Green SuperMix ROX (9505502K, Quantabio). RT-qPCR results were analyzed by the ^ΔΔ^Ct method and normalized to *Gapdh/GAPDH* or *Tbp*.

### RNA ISH (RNAscope).

Chromogenic RNA ISH was performed with bacterial *DapB* (negative control) (no. 310043), human *PPIB* (positive control) (no. 313901), mouse *Ppib* (positive control) (no. 313911), human *SELENOP* (no. 512831), or mouse *Selenop* (no. 549611) RNAscope probes (all from Advanced Cell Diagnostics) and RNAscope 2.5 HD – BROWN reagents (no. 322300, Advanced Cell Diagnostics) per the manufacturer’s protocol.

### scRNA-Seq data analysis and visualization.

Gut Cell Atlas scRNA-Seq expression data (24) was explored at https://www.gutcellatlas.org Human colorectal polyp/cancer scRNA-Seq data (27, 29) (HTA10, HTA11) are publicly available through the Human Tumor Atlas Network (https://data.humantumoratlas.org). Human CRC scRNA-Seq data (30) (GSE178341) are publicly available through NCBI’s Gene Expression Omnibus (GEO) (https://www.ncbi.nlm.nih.gov/geo/). These scRNA-Seq data sets were analyzed in Python using scanpy, pandas, and numpy packages as previously described (27). Briefly, raw scRNA-Seq counts were normalized to the median library size, log-like transformed with Arcsinh, and *z* score–standardized per gene. CytoTRACE analysis (28) was conducted as previously described (27).

Polyp, normal, and cancer tissue data sets from (27) were integrated with the Single-Cell Regulatory Network Inference and Clustering (SCENIC) pipeline (65, 66). From the SCENIC-derived, *z* score–standardized AUCell values, the “scanpy.tl.umap” function was used to compute UMAP coordinates, 50–principal component decompositions with no feature selection, and k-nearest-neighbor graphs, with k equal to the square root of the number of cells projected. The UMAP visualization for the data set from ref. 29 was produced by the same procedure but with normalized count values. Strip plots were generated from downsampled data of the corresponding bar plots, to keep cell number for all data set categories equal to the cell number of the smallest category.

### Human enteroid culture.

Human jejunal organoids were a gift from James Goldenring (Vanderbilt University, Nashville, Tennessee, USA). These enteroids were established from deidentified tissue collected at VUMC and provided by the Western Division of the Cooperative Human Tissue Network (CHTN) in accordance with the IRB of VUMC. Enteroids were refed with Intesticult Organoid Growth Medium (06010, STEMCELL Technologies) every 4 days. For ELISA experiments, enteroids were refed every 2–3 days with media described in Supplemental Table 3. Enteroids were split and replated every 7–10 days as described below.

Enteroids were collected by centrifugation at 200*g* for 5 minutes at 4°C, gently sheared approximately 20 times by pipetting, then centrifuged again as above. Enteroid fragments were resuspended in growth factor–reduced (GFR) Matrigel (354230, Corning), plated in 4 approximately 12 μL plugs per well, incubated at 37°C for 30 minutes, and fed with 500 μL Intesticult Organoid Growth Medium.

### ELISAs.

Human enteroid conditioned media (3–4 mL) were concentrated using Amicon Ultra-4 10 kDa centrifugal filters (MilliporeSigma, UFC801024) to yield a final volume of approximately 500 μL. 293 STF and RKO-dCas9-VPR cell lines were cultured to approximately 50% confluence in 6-well plates, then refed with serum-free DMEM (11995065, Gibco, Thermo Fisher Scientific) for 96 hours. SELENOP sandwich ELISAs were performed with N22 and N11 capture and detection antibodies, respectively, as described previously (67).

### Murine tumorigenesis protocol.

*Lrig1-CreERT2/^+^* (*Lrig1^tm1.1(cre/ERT2)Rjc^*/J, 018418, The Jackson Laboratory); *Apc^fl/+^*(*Apc^tm1Tyj^*/J, 009045, The Jackson Laboratory) and *Selenop^–/–^* (*Selenop^tm1Rfb^*/J, 008201, The Jackson Laboratory) mice were previously generated (31, 32, 68) and backcrossed with mice on a C57BL/6J background. *Lrig1-CreERT2/^+^ Apc^fl/fl^ Selenop^+/–^* mice were bred with *Selenop^+/–^* mice to generate female and male littermates for experiments. All mice were housed under a 12-hour dark/12-hour light cycle and provided a selenium-supplemented (1.0 mg selenium/kg) defined diet (Envigo) ad libitum. Bedding from all cages was mixed and redistributed 2 weeks before experiments and every 2 weeks thereafter to minimize microbiome variation.

Cohorts of 8- to 10-week-old *Lrig1-CreERT2/^+^ Apc^fl/+^ Selenop^+/+^*, *Selenop^+/–^*, and *Selenop^–/–^* mice were administered 3 daily i.p. injections of 2 mg tamoxifen (T5648, MilliporeSigma) dissolved in corn oil (Mazola). Mice were colonoscopically monitored for tumors on days 50, 64, 78, and 92 after the initial tamoxifen injection and then euthanized on day 100 (35) by experimenters blinded to their genotype. Small intestine and colon tissue was macroscopically imaged and analyzed and then Swiss-rolled and formalin-fixed for unstained and H&E-stained slide preparation by the VUMC Translational Pathology Shared Resource (TPSR). Colon tumor volume was calculated from length (L) and width (W) measurements with the formula W^2^ × L/2 (69). H&E-stained slides were examined for dysplasia severity by a gastrointestinal pathologist blinded to genotype.

### Murine tumoroid culture.

Tumoroids were established from *Apc^ΔIE/+^ Selenop^+/+^* and *Selenop^–/–^* mice as described previously (22). Tumoroids were refed with basal media supplemented with 20% R-spondin–conditioned media and 10% Noggin-conditioned media every 3 days. Tumoroids were split and replated every 7–10 days as described below.

Tumoroids were collected by centrifugation at 200*g* for 5 minutes at 4°C, gently sheared twice through a 25 gauge needle, and then centrifuged again as above. For subculturing and expansion, tumoroid fragments were resuspended in GFR Matrigel and plated in 50 μL plugs. For enzymatic dissociation experiments, tumoroids were resuspended in TrypLE Express (12604013, Gibco, Thermo Fisher Scientific) with 10 μM Y-27632 (1254, Tocris Bioscience) and 50 μg/mL DNase I (D5025, MilliporeSigma), incubated at 37°C for 3 minutes, and filtered through a 70 μm cell strainer. Enzymatic dissociation was halted by addition of PBS (without calcium or magnesium) and centrifugation as above. Tumoroid cells were then resuspended in GFR Matrigel and plated at a density of 5,000 live cells per 50 μL plug. Tumoroid fragments per cells were incubated at 37°C for 30 minutes, then fed with 500 μL basal media supplemented with 20% (v/v) R-spondin–conditioned media and 10% (v/v) Noggin-conditioned media.

### Murine tumoroid image quantification.

Tumoroids were imaged after 5 days with an EVOS FL2 Auto Imaging System (Thermo Fisher Scientific). The tumoroid number was quantified in Image (NIH) (70) by an experimenter blinded to the genotype.

### Cell lines and maintenance.

293T (CRL3216), Hep G2 (HB-8065), and RKO (CRL2577) cells were purchased from the American Type Culture Collection (ATCC), which confirms cell line identity by short tandem repeat analysis. 293 Super TOPFlash (293 STF) cells were a gift from Ethan Lee (Vanderbilt University, Nashville, Tennessee, USA) and Jeremy Nathans (Johns Hopkins University, Baltimore, Maryland, USA) (21, 37). Although 293 STF cells were not authenticated in our laboratory, they demonstrate the expected G418 resistance and WNT-induced TOPFlash reporter activity. 293T-FLAG-LRP6 cells were a gift from Victoria Ng and Ethan Lee (both from Vanderbilt University, Nashville, Tennessee, USA). MC38 cells were a gift from Barbara Fingleton (Vanderbilt University, Nashville, Tennessee, USA). YAMC cells, generated and as described in ref. 71, were obtained from the VUMC Digestive Disease Research Center (DDRC) GI Organoid Subcore.

293 STF, 293T, Hep G2, MC38, and RKO cell lines were maintained in DMEM (11995065, Gibco, Thermo Fisher Scientific) supplemented with 10% (v/v) FBS (07068085, Avantor) and 1% (v/v) penicillin/streptomycin (15140122, Gibco, Thermo Fisher Scientific), and cultured at 37°C in 5% CO_2_. YAMC cell lines were maintained in RPMI 1640 Medium (61870036, Gibco, Thermo Fisher Scientific) supplemented with 10% (v/v) FBS, 1% (v/v) penicillin/streptomycin, and 10 U/mL recombinant mouse IFN-γ (485MI100/CF, R&D Systems), and cultured at 33°C in 5% CO_2_. All cells used for experiments were passaged fewer than 15 times and regularly tested for mycoplasma contamination with a Mycoplasma PCR Detection Kit (G238, Applied Biological Materials [abm]).

### Lentiviral transduction.

293T cells were cultured to approximately 50% confluence in 10 cm plates and then cotransfected with 1 μg pMD2.G (12259, Addgene) envelope plasmid, 1 μg psPAX2 (12260, Addgene) packaging plasmid, and 2 μg 7TFP (24308, Addgene), lenti dCAS-VP64_Blast (61425, Addgene), lentiGuide-Puro-NONTARGET (the present study), lentiGuide-Puro-hSELENOP (the present study), lentiGuide-Puro-mSELENOP (the present study), pLV-mCherry (VectorBuilder), pLV-hSELENOP (VectorBuilder), pLX304-V5-mSELENOP (the present study), or pLX304-V5-mSELENOP_Δ258-299 (this paper) using polyethylenimine (24314, Polysciences). Cells were refed 16 hours after transfection, and lentiviral supernatants were passed through 0.45 μm filters 48 hours later. Target cells were transduced overnight in filtered lentivirus containing 5 μg/mL polybrene (TR1003G, MilliporeSigma). For tumoroids, filtered lentiviral supernatants were concentrated with Lenti-X Concentrator (631232, Takara Bio) according to the manufacturer’s protocol. Target tumoroids were transduced for 4 hours in concentrated lentivirus with 8 μg/mL polybrene and 10 μM Y-27632. Forty-eight hours later, cells and tumoroids were selected with the following concentrations of puromycin (P8833, MilliporeSigma) or blasticidin (ant-bl-05, InvivoGen): 1 μg/mL puromycin (293 STF, MC38, and RKO cells), 3 μg/mL puromycin (tumoroids), 5 μg/mL puromycin (YAMC cells), 5 μg/mL blasticidin (tumoroids), or 10 μg/mL blasticidin (YAMC STF cells).

### CRISPRa cell line generation.

RKO and MC38 cells were cultured to approximately 50% confluence in 10 cm plates and then cotransfected with 1 μg pCMV-HA-m7pB (72) transposase plasmid and 2.5 μg PB-TRE-dCas9-VPR (63800, Addgene) transposon plasmid using Lipofectamine 2000 (11668019, Invitrogen, Thermo Fisher Scientific). Cells were selected with 100 μg/mL hygromycin B (10687010, Gibco, Thermo Fisher Scientific) 72 hours later. *SELENOP* or *Selenop* promoter–targeted CRISPRa sgRNAs were designed with the CRISPick tool (Broad Institute). The top-4-ranked candidates were ordered as oligonucleotides (Integrated DNA Technologies), cloned into lentiGuide-Puro (52963, Addgene) as described previously (73), and sequence-verified by GENEWIZ with U6 GENEWIZ universal primers. As lentiGuide-Puro-hSELENOP_3 and lentiGuide-Puro-mSELENOP_3 yielded the greatest *SELENOP/Selenop* overexpression in RKO- and MC38-dCas9-VPR cells, respectively, these sgRNAs were used for subsequent experiments. All sgRNA sequences are listed in Supplemental Table 4.

### WNT3A treatments.

293 STF and RKO cell lines were treated with 400 ng/mL and 200 ng/mL recombinant human WNT3A (rhWNT3A) (5036WNP10/CF, R&D Systems), respectively, for 16 hours prior to TOPFlash assays. MC38 and YAMC cell lines were treated with 35 ng/mL and 100 ng/mL recombinant mouse WNT3A (rmWNT3A) (1324WN010/CF, R&D Systems), respectively, for 16 hours prior to TOPFlash assays.

### TOPFlash reporter assays.

293 STF, RKO STF, and YAMC STF cell lines were seeded in 12-well plates (100,000 cells/well). Thirty-two hours after plating, 293 STF and RKO STF cell lines were treated with or without rhWNT3A (5036WNP10/CF, R&D Systems) and 0, 20, 40, 60, 80, or 100 ng/mL purified human SELENOP for 16 hours, whereas YAMC STF cell lines were treated with or without rmWNT3A (1324WN010/CF, R&D Systems) for 16 hours. Cells were lysed in 1× Glo Lysis Buffer (E2661, Promega), and lysates were mixed 1:1 with Steady-Glo luciferase reagent (E2510, Promega) or CellTiter-Glo luminescent cell viability reagent (G7570, Promega). Luminescence was measured with a GloMax Discover microplate reader (Promega). Steady-Glo readings were normalized to CellTiter-Glo readings to account for cell viability.

RKO-dCas9-VPR and MC38-dCas9-VPR cell lines were seeded in 12-well plates (50,000 cells/well). Twenty-four hours later, cells were cotransfected with 0.50 μg M50 Super 8x TOPFlash reporter plasmid (12456, Addgene) and 0.05 μg pRL-TK control reporter plasmid (E2241, Promega) using Lipofectamine 2000. Forty-eight hours later, cells were treated with or without WNT3A for 16 hours. Cells were lysed in Dual-Glo luciferase reagent (E2920, Promega), luminescence was measured with a GloMax Discover microplate reader (Promega), Dual-Glo Stop & Glo reagent (E2920, Promega) was added, and luminescence was measured again. Dual-Glo readings were normalized to Stop & Glo readings to control for transfection efficiency.

### FLAG IPs.

293T cells were cultured to approximately 50% confluence in 10 cm plates and then cotransfected with 2 μg pcDNA6-N-3XFLAG-Lrp6 (123595, Addgene) and 2 μg mSELENOP plasmids (ref. 62 and the present study) with polyethylenimine. Forty-eight hours later, cells were incubated on ice for 10 minutes in FLAG IP Lysis Buffer (L3412, MilliporeSigma) with phosphatase inhibitor cocktail 2 (P5726, MilliporeSigma), phosphatase inhibitor cocktail 3 (P0044, MilliporeSigma), and protease inhibitor cocktail (P8340, MilliporeSigma), and then transferred into microcentrifuge tubes and centrifuged at 16,000*g* for 10 minutes at 4°C. Supernatant protein concentrations were quantified with a BCA Protein Assay Kit (23225, Pierce, Thermo Fisher Scientific). Total protein (2 mg) was used for IP with ANTI–FLAG M2 Affinity Gel (A2220, MilliporeSigma) according to the manufacturer’s protocol. Bound proteins were eluted with 150 ng/μL 1x FLAG Peptide (F3290, MilliporeSigma) at 4°C for 30 minutes.

### Heparin and sodium chlorate treatments.

293T or 293T-FLAG-LRP6 cells were cultured to approximately 50% confluence in 10 cm plates and then treated with 1 mg/mL heparin (H3393, MilliporeSigma) or 50 mM sodium chlorate (244147, MilliporeSigma) for 48 hours prior to FLAG IPs.

### SELENOP-conditioned media preparation.

Hep G2 cells were seeded in 10 cm plates (3,000,000 cells/plate). After 48 hours, SELENOP-conditioned media were collected and centrifuged at 500*g* for 5 minutes at 4°C.

### Cell-surface biotinylation and isolation experiments.

293T cells were cultured to approximately 80% confluence in 10 cm plates and then treated with 3 mL complete DMEM or SELENOP-conditioned media for 2 hours. Cells were biotinylated and lysed with a Cell Surface Biotinylation and Isolation Kit (A44390, Pierce, Thermo Fisher Scientific) per the manufacturer’s protocol. Lysate concentrations were quantified with a BCA Protein Assay Kit. Equal amounts of total protein were used for pulldown with NeutrAvidin Agarose (29200, Pierce, Thermo Fisher Scientific), and bound proteins were eluted with DTT (A39255, Pierce, Thermo Fisher Scientific).

### Plasmid construction.

pCMV6-V5-mSELENOP (full-length) and pCMV6-mSELENOP (tU3, tU4, tU5, tU6, tU7, and tU9) constructs were a gift from Suguru Kurokawa (Osaka Ohtani University, Tondabayashi, Osaka, Japan) and are described elsewhere (62). pCMV6-V5-mSELENOP tU1, tU2, tU3, tU4, Δ258-267, Δ268-277, Δ278-287, Δ288-299, and Δ258-299 plasmids were generated via round-the-horn PCR as described previously (74), using the primers listed in Supplemental Table 5. All pCMV6-V5-mSELENOP constructs were sequence verified by GENEWIZ with T7 and M13R GENEWIZ universal primers.

pLX304-V5-mSELENOP plasmids (full-length and Δ258-299) were generated by Gateway cloning (75) (Thermo Fisher Scientific) per the manufacturer’s protocol. Briefly, V5-mSELENOP was flanked by attB sites via PCR amplification from pCMV6-V5-mSELENOP (full-length or Δ258-299) using the primers listed in Supplemental Table 5 and Q5 Hot Start High-Fidelity 2X Master Mix (M0494S, New England BioLabs). attB-flanked PCR products were purified with the QIAquick PCR Purification Kit (28104, QIAGEN) prior to BP reactions with Gateway pDONR221 (12536017, Invitrogen, Thermo Fisher Scientific) using Gateway BP Clonase II Enzyme mix (11789020, Invitrogen, Thermo Fisher Scientific). attL/attR recombination (LR) reactions were then performed with the attB/attP recombination (BP) reactions and pLX304 (25890, Addgene) using Gateway LR Clonase II Enzyme mix (11791020, Invitrogen, Thermo Fisher Scientific). All pLX304-V5-mSELENOP constructs were sequence verified by Plasmidsaurus.

### Immunoblot analysis.

Protein samples were diluted in 4× Laemmli Sample Buffer (1610747, Bio-Rad) with 6% (v/v) 2-mercaptoethanol (M6250, MilliporeSigma) and then incubated at 95°C for 5 minutes. Protein (40–80 μg) was loaded into each lane of a 4%–20% Mini-PROTEAN TGX Precast Protein Gel (4561094, Bio-Rad), alongside Precision Plus Protein Dual Color Standards (1610374, Bio-Rad) for SDS-PAGE. SDS-PAGE–separated proteins were transferred onto a 0.45 μm nitrocellulose membrane (NBA085C001EA, PerkinElmer), blocked with Intercept (TBS) Blocking Buffer (927-60001, LI-COR) at room temperature for 30 minutes, and then probed with primary antibodies diluted in 50% Intercept (TBS) Blocking Buffer/50% TBS with 0.1% (v/v) Tween-20 (P1379, MilliporeSigma) (TBS-T) at 4°C overnight. The primary antibodies used included: rabbit anti–β-tubulin (1:2,000, 2146, Cell Signaling Technology); mouse anti-FLAG (1:1,000, F1804, MilliporeSigma); rabbit anti-LRP6 (1:1,000, 2560, Cell Signaling Technology); rabbit anti-LRP6 (1:1,000, 3395, Cell Signaling Technology); rabbit anti–Na^+^/K^+^-ATPase (1:1,000, 3010, Cell Signaling Technology); mouse anti-SELENOP (1:1,000, N11, Vanderbilt Antibody and Protein Resource); rabbit anti-SELENOP (1:1,000, Proteintech Group, a gift from Suguru Kurokawa, Osaka Ohtani University, Tondabayashi, Osaka, Japan) (76); mouse anti-V5 (1:1,000, ab27671, Abcam); and rabbit anti-V5 (1:1,000, 13202, Cell Signaling Technology). Membranes were washed with TBS-T and then probed with IRDye 680LT Goat anti–mouse IgG (1:10,000, 92668020, LI-COR) and IRDye 800CW goat anti–rabbit IgG (1:10,000, 92632211, LI-COR) secondary antibodies diluted in TBS-T at room temperature for 30 minutes. Membranes were washed again with TBS-T, imaged with an Odyssey Clx near-infrared fluorescence imaging system (LI-COR), and quantified with Image Studio (LI-COR). Densitometric values for the proteins of interest were normalized to those of their corresponding loading controls.

### Figure design.

The schematics and Graphical Abstract were created with Biorender.com under the Vanderbilt University School of Medicine Basic Sciences institutional license. Portions of the Graphical Abstract were adapted from “Wnt Signaling Pathway Activation and Inhibition” by Biorender.com (2023), retrieved from https://app.biorender.com/biorender-templates All other figures were designed in Inkscape (version 1.2.2).

### Statistics.

Statistical analyses for scRNA-Seq data were performed in Python with scipy.stats and seaborn packages. All other statistical analyses were performed in GraphPad Prism (version 9.5.1, GraphPad Software). A *P* value of less than 0.05 was considered statistically significant.

### Study approval.

All animal experiments were carried out in accordance with protocols approved by the IACUC of VUMC. All human tissues were provided by the Western Division of the CHTN in accordance with the VUMC IRB.

### Data availability.

Values for all data points found in graphs can be found in the supplemental supporting data values file.

## Author contributions

JMP designed and performed experiments, analyzed data, and wrote the manuscript. REB, NJB, APO, and SPS performed experiments. ZC and KSL analyzed scRNA-Seq data. MKW performed histological analyses and provided pathological expertise. SAA quantified tumoroid images. SK, VHN, JJT, and JJ generated reagents. JAG, EL, YAC, KSL, SPS, and CSW provided intellectual contributions to the experimental design and analysis. All authors edited and approved the manuscript.

## Supplementary Material

Supplemental data

Supporting data values

## Version 1 05/11/2023

In-Press Preview

## Version 2 07/03/2023

Electronic publication
