# Supplementary material for: SELENOP modifies sporadic colorectal carcinogenesis and WNT signaling activity through LRP5/6 interactions
Source: J Clin Invest. 2023 Jul 3;133(13):e165988. doi: 10.1172/JCI165988 (PMC10313376; doi:10.1172/JCI165988)
Supplement: Supplemental data [file jci-133-165988-s038.pdf]

## SUPPLEMENTAL METHODS

*Immunoblots*

Additional primary antibodies included rabbit anti-APC (1:1000, sc-7930, Santa Cruz Biotechnology), rabbit anti-GAPDH (1:3000, 5174, Cell Signaling Technology), rabbit anti-GPX1 (1:1000, SAB5700925, Sigma-Aldrich), and rabbit anti-GPX2 (1:1000, ab137431, abcam).

*Small intestine tumor area measurements*

Well-oriented, H&E-stained small intestine adenomas were imaged at 10x magnification and measured in ImageJ (1) by an experimenter blinded to genotype.

*Murine enteroid culture*

Enteroids were established and cultured as previously described (2).

*Human tumoroid culture*

Human tumoroids were established and cultured as previously described (3). Known clinical characteristics are described in Table S5.

*SELENOP treatments*

Human tumoroids were treated with 0 or 500 ng/mL purified human SELENOP for five days prior to RNA extraction. 293 STF cells were treated with 0 or 100 ng/mL purified human SELENOP for 16 hours prior to TOPFlash assays.

*Small interfering RNA (siRNA) transfections*

293 STF cells were seeded in 6-well or 12-well plates (300,000 or 100,000 cells/well, respectively). Twenty-four hours later, cells were transfected with 100 nM control siRNA-A (sc37007, Santa Cruz Biotechnology) or pooled *APC* siRNAs (sequences published in (4), Dharmacon) using Lipofectamine® RNAiMAX (13778075, Invitrogen).

*Proximity ligation assay*

293T cells were cultured to ~10% confluency in 8-well chamber slides (PEZGS0816, Millipore), then transfected with 0.1 µg pcDNA6-N-3XFLAG-Lrp6 (123595, Addgene) and 0.1 µg pCMV6-V5-mSELENOP (5) plasmids using polyethylenimine (24314, Polysciences, Inc.). After 48 hours, cells were fixed in 3% (w/v) paraformaldehyde (158127, Sigma-Aldrich), briefly washed in PBS with 10 mM glycine

(G36050, Research Products International), and permeabilized in PBS with 0.2% (v/v) Triton™ X-100 (T8787, Sigma-Aldrich). Proximity ligation assays were then performed with mouse anti-β-catenin (1:500, 610154, BD Biosciences), rabbit anti-α-catenin (1:500, C2081, Sigma-Aldrich), mouse IgG1 (1:500, 5415, Cell Signaling Technology), rabbit IgG (1:500, 3900, Cell Signaling Technology), mouse anti-FLAG® M2 (1:500, F1804, Sigma-Aldrich), and rabbit anti-V5 (1:500, 13202, Cell Signaling Technology) antibodies and the Duolink® In Situ Red Starter Kit Mouse/Rabbit (DUO92101, Sigma-Aldrich) per the manufacturer's protocol. Slides were imaged with a Nikon Eclipse E800 upright microscope and NIS-Elements BR software.

*Plasmids*

pReceiver-M14-mLRP5-3XFLAG was purchased from GeneCopoeia (EX-Mm34003-M14).

## SUPPLEMENTAL REFERENCES


1. Schneider CA, Rasband WS, Eliceiri KW. NIH Image to ImageJ: 25 years of image analysis. *Nat Methods* 2012;9(7):671–675.

2. Barrett C, Short S, Choksi Y, Williams C. Whole-mount Enteroid Proliferation Staining. *Bio-protocol* 2016;6(12). doi:10.21769/bioprotoc.1837

3. Short SP et al. Serine Threonine Kinase 17A maintains the epithelial state in colorectal cancer cells. *Mol Cancer Res* 2019;17(4):molcanres.0990.2018.

4. Saito-Diaz K et al. APC Inhibits Ligand-Independent Wnt Signaling by the Clathrin Endocytic Pathway. *Dev Cell* 2018;44(5):566-581.e8.

5. Kurokawa S, Bellinger FP, Hill KE, Burk RF, Berry MJ. Isoform-specific Binding of Selenoprotein P to the β-Propeller Domain of Apolipoprotein E Receptor 2 Mediates Selenium Supply. *J Biol Chem* 2014;289(13):9195–9207.

## SUPPLEMENTAL TABLES

| Gene Name | Assay ID |
|---|---|
| *Dio1* | Mm00839358_m1 |
| *Dio2* | Mm00515664_m1 |
| *Dio3* | Mm00548953_s1 |
| *Gapdh* | Mm99999915_g1 |
| *Gpx1* | Mm00656767_g1 |
| *Gpx2* | Mm00850074_g1 |
| *Gpx3* | Mm00492427_m1 |
| *Gpx4* | Mm00515041_m1 |
| *Msrb1* | Mm00489121_m1 |
| *Selenof* | Mm00474111_m1 |
| *Selenoh* | Mm01335355_g1 |
| *Selenoi* | Mm01210813_m1 |
| *Selenok* | Mm00785961_s1 |
| *Selenom* | Mm00459806_m1 |
| *Selenon* | Mm01188435_m1 |
| *Selenoo* | Mm00662744_m1 |
| *Selenop* | Mm00486048_m1 |
| *Selenos* | Mm01318786_m1 |
| *Selenot* | Mm01615823_m1 |
| *Selenov* | Rn01475733_m1 |
| *Selenow* | Mm01268252_m1 |
| *Sephs2* | Mm00545980_s1 |
| *Tbp* | Mm00446973_m1 |
| *Txnrd1* | Mm00443675_m1 |
| *Txnrd2* | Mm00496766_m1 |
| *Txnrd3* | Mm00462552_m1 |

**Table S1. TaqMan™ RT-qPCR probes.**

| Gene Name | Primer Designations | Primer Sequences | Reference |
| --- | --- | --- | --- |
| *Axin2* | mAxin2_RT_F<br>mAxin2_RT_R | 5’ TGACTCTCCTTCCAGATCCCA 3’<br>5’ TGCCCACACTAGGCTGACA 3’ | Short et al. (2019) *Oncogene*. |
| *AXIN2* | hAXIN2_RT_F<br>hAXIN2_RT_R | 5’ CAACACCAGGCGGAACGAA 3’<br>5’ GCCCAATAAGGAGTGTAAGGACT 3’ | Thompson et al. (2019) *Carcinogenesis* |
| *Gapdh* | mGapdh_RT_F<br>mGapdh_RT_R | 5’ CCGCATCTTCTTGTGCA 3’<br>5’ CGGCCAAATCCGTTCA 3’ | Short et al. (2019) *Oncogene*. |
| *GAPDH* | hGAPDH_RT_F<br>hGAPDH_RT_R | 5’ GGCCTCCAAGGAGTAAGACC 3’<br>5’ AGGGGTCTACATGGCAACTG 3’ | Thompson et al. (2019) *Carcinogenesis* |
| *Lgr5* | mLgr5_RT_F<br>mLgr5_RT_R | 5’ CCAATGGAATAAAGACGACGGCAACA 3’<br>5’ GGGCCTTCAGGTCTTCCTCAAAGTCA 3’ | Luong-Gardiol et al. (2019) *Cancer Cell.* |
| *LGR5* | hLGR5_RT_F<br>hLGR5_RT_R | 5’ GAGTTACGTCTTGCGGGAAAC 3’<br>5’ TGGGTACGTGTCTTAGCTGATTA 3’ | Liao et al. (2020) *Stem Cell Rep.* |
| *Sox9* | mSox9_RT_F<br>mSox9_RT_R | 5’ GAGCCGGATCTGAAGAGGGA 3’<br>5’ GCTTGACGTGTGGCTTGTTC 3’ | Wang et al. (2020) *Cancer Cell.* |
| *SOX9* | hSOX9_RT_F<br>hSOX9_RT_R | 5’ AGCGAACGCACATCAAGAC 3’<br>5’ CTGTAGGCGATCTGTTGGGG 3’ | Li et al. (2015) *PLoS One.* |

**Table S2. SYBR Green RT-qPCR primers.**

<table>
<tr><th>Condition</th><th>Basal Media</th><th>Supplements</th><th>Additives</th></tr>
<tr><td>Stem cell</td><td rowspan="4">Advanced DMEM/F12<br>(12634010, Gibco)<br><br>1X B-27™ Supplement<br>(17504044, Gibco)<br><br>1X GlutaMAX™<br>(35050061, Gibco)<br><br>1X N-2™ Supplement<br>(17502048, Gibco)<br><br>1 mM HEPES<br>(15630080, Gibco)<br><br>2% (v/v) penicillin/streptomycin<br>(15140122, Gibco)</td><td rowspan="4">20% (v/v) R-spondin-conditioned media (from R-spondin-expressing cells gifted by Dr. Jeff Whitsett, The University of Cincinnati, Cincinnati, OH)<br><br>10% (v/v) Noggin-conditioned media (from Noggin-expressing cells gifted by Dr. G.R. van den Brink, Amsterdam, NL)</td><td>None</td></tr>
<tr><td>Enterocyte</td><td>2 μM IWP 2<br>(3533, Tocris Bioscience)<br><br>2 mM valproic acid<br>(P4543, Sigma-Aldrich)</td></tr>
<tr><td>Paneth cell</td><td>3 μM CHIR 99021<br>(4423, Tocris Bioscience)<br><br>10 μM DAPT<br>(2634, Tocris Bioscience)</td></tr>
<tr><td>Goblet cell</td><td>10 μM DAPT<br>(2634, Tocris Bioscience)<br><br>2 μM IWP 2<br>(3533, Tocris Bioscience)</td></tr>
</table>

**Table S3. Human enteroid media components.**

| sgRNA Name | sgRNA Designations | sgRNA Sequences |
|---|---|---|
| NONTARGET | NONTARGET_CRa_F_1<br>NONTARGET_CRa_R_1 | 5’ CACCGGACCTTCATTGAAGAAAAGC 3’<br>5’ AAACGCTTTTCTTCAATGAAGGTCCGGTGC 3’ |
| hSELENOP_1 | hSELENOP_CRa_F_1<br>hSELENOP_CRa_R_1 | 5’ CACCGGGAAGGGCTAAGGGTAAACA 3’<br>5’ AAACTGTTTACCCTTAGCCCTTCCCGGTGC 3’ |
| hSELENOP_2 | hSELENOP_CRa_F_2<br>hSELENOP_CRa_R_2 | 5’ CACCGGTTTGGGAAAGAAGGCAACT 3’<br>5’ AAACAGTTGCCTTCTTTCCCAAACCGGTGC 3’ |
| hSELENOP_3 | hSELENOP_CRa_F_3<br>hSELENOP_CRa_R_3 | 5’ CACCGTTCTTTCCCAAACTATAACA 3’<br>5’ AAACTGTTATAGTTTGGGAAAGAACGGTGC 3’ |
| hSELENOP_4 | hSELENOP_CRa_F_4<br>hSELENOP_CRa_R_4 | 5’ CACCGTGGGAAAGAAGGCAACTTGG 3’<br>5’ AAACCCAAGTTGCCTTCTTTCCCACGGTGC 3’ |
| mSELENOP_1 | mSELENOP_CRa_F_1<br>mSELENOP_CRa_R_1 | 5’ CACCGACTTTGGACTGCACCTCAGA 3’<br>5’ AAACTCTGAGGTGCAGTCCAAAGTCGGTGC 3’ |
| mSELENOP_2 | mSELENOP_CRa_F_2<br>mSELENOP_CRa_R_2 | 5’ CACCGCTGCATTTGCAAGGTCGCAG 3’<br>5’ AAACCTGCGACCTTGCAAATGCAGCGGTGC 3’ |
| mSELENOP_3 | mSELENOP_CRa_F_3<br>mSELENOP_CRa_R_3 | 5’ CACCGGCTGAGGCAGTACTTACTGA 3’<br>5’ AAACTCAGTAAGTACTGCCTCAGCCGGTGC 3’ |
| mSELENOP_4 | mSELENOP_CRa_F_4<br>mSELENOP_CRa_R_4 | 5’ CACCGGTTGTTTACCTCGCCCTCTG 3’<br>5’ AAACCAGAGGGCGAGGTAAACAACCGGTGC 3’ |

**Table S4. sgRNA sequences.**

| Construct Name | Primer Designations | Primer Sequences |
|---|---|---|
| pCMV6-V5-mSELENOP_tU1 | mSELENOP_tU_F | 5’ TACGACTAAGCAAGAATGGAGTACA GAATTAAGTG 3’ |
| | mSELENOP_tU1_R | 5’ TAAGCTGGCTTGAAGAAGAGCAACC ACTGTCACTT 3’ |
| pCMV6-V5-mSELENOP_tU2 | mSELENOP_tU_F | 5’ TACGACTAAGCAAGAATGGAGTACA GAATTAAGTG 3’ |
| | mSELENOP_tU2_R | 5’ TAAGCTCTCTAAGTGACCCTGCCTG TGCTGGCCCC 3’ |
| pCMV6-V5-mSELENOP_tU3 | mSELENOP_tU_F | 5’ TACGACTAAGCAAGAATGGAGTACA GAATTAAGTG 3’ |
| | mSELENOP_tU3_R | 5’ TAAGAGCTTCCTCTGGGCAAGTGAA AGGTGCAAGC 3’ |
| pCMV6-V5-mSELENOP_tU4 | mSELENOP_tU_F | 5’ TACGACTAAGCAAGAATGGAGTACA GAATTAAGTG 3’ |
| | mSELENOP_tU4_R | 5’ TAAAGCAATTGCAGACCCTGACTTC TCAAATATGA 3’ |
| pCMV6-V5-mSELENOP_Δ258-267 | mSELENOP_d258-267_F | 5’ TGTAAGTTGTCTAAGGAGTCCGAGG CAGCCCCCAG 3’ |
| | mSELENOP_d258-267_R | 5’ GAGCTTCCTCTGGGCAAGTGAAAGG TGCAAGCCTT 3’ |

| | | |
|---|---|---|
| pCMV6-V5-mSELENOP_Δ268-277 | mSELENOP_d268-277_F<br>mSELENOP_d268-277_R | 5’ CCCAGCAGCTGCTGCTGTCACTGCC GCCACCTCAT 3’<br>5’ CAGGAGCTGGTTGATGCACCCCCTT CGACAGAGCT 3’ |
| pCMV6-V5-mSELENOP_Δ278-287 | mSELENOP_d278-287_F<br>mSELENOP_d278-287_R | 5’ TTTGAGAAGTCAGGGTCTGCAATTG CTTGTCAGTG 3’<br>5’ GGCTGCCTCGGACTCCTTAGACAAC TTACACAGGA 3’ |
| pCMV6-V5-mSELENOP_Δ288-299 | mSELENOP_d288-299_F<br>mSELENOP_d288-299_R | 5’ CAGTGTGCGGAAAACCTCCCATCCT 3’<br>5’ TATGAGGTGGCGGCAGTGACAGCAG 3’ |
| pCMV6-V5-mSELENOP_Δ258-299 | mSELENOP_d288-299_F<br>mSELENOP_d258-267_R | 5’ CAGTGTGCGGAAAACCTCCCATCCT 3’<br>5’ GAGCTTCCTCTGGGCAAGTGAAAGG TGCAAGCCTT 3’ |
| pLX304-V5-mSELENOP | attB1-mSELENOP_F<br>attB2-mSELENOP_R | 5’ GGGGACAAGTTTGTACAAAAAAGCA GGCTTCACCATGTGGAGAAGCCTAG GGCTTGCC 3’<br>5’ GGGGACCACTTTGTACAAGAAAGCT GGGTCTTAGTTTGAATGACATTTACA CTT 3’ |
| pLX304-V5-mSELENOP_ Δ258-299 | attB1-mSELENOP_F | 5’ GGGGACAAGTTTGTACAAAAAAGCA GGCTTCACCATGTGGAGAAGCCTAG GGCTTGCC 3’ |

| | | |
|---|---|---|
| | attB2-mSELENOP_R | 5’ GGGGACCACTTTGTACAAGAAAGCT GGGTCTTAGTTTGAATGACATTTACA CTT 3’ |

**Table S5. PCR primers for plasmid construction.**

| **Line** | **Location** | **Age** | **Race** | **Sex** | **Stage** | **Dysplasia** | **Known Mutations** | **MSI/MSS** | **CMS** |
|---|---|---|---|---|---|---|---|---|---|
| 32385 | Right | 61 | Black | Female | T3N0 | HGD | | MSI | 1/3 |
| 35349 | Sigmoid | 57 | White | Female | T3N0 | HGD | $KRAS^{G12D}$, $TP53^{R248W}$ | MSS | 2/4 |
| 40299 | Sigmoid | 67 | White | Female | T3N1b | LGD | | MSS | 4 |
| 82742 | Right | 79 | Black | Male | T4aN2b | HGD | | MSS | 2 |

**Table S6. Clinical characteristics of human colon tumors from which tumoroids were established.** Stage and dysplasia were determined by the attending pathologist. All patients were treatment-naïve. For line 35349, mutational analysis was performed on biopsy tissue prior to resection. Lines 32385, 40299, and 82742 were not subjected to further mutational analysis. Microsatellite instability was analyzed by PCR and IHC per clinical standard of care. CMS: consensus molecular subtype, HGD: high-grade dysplasia, LGD: low-grade dysplasia, MSI: microsatellite instability, MSS: microsatellite stable.

## SUPPLEMENTAL FIGURES

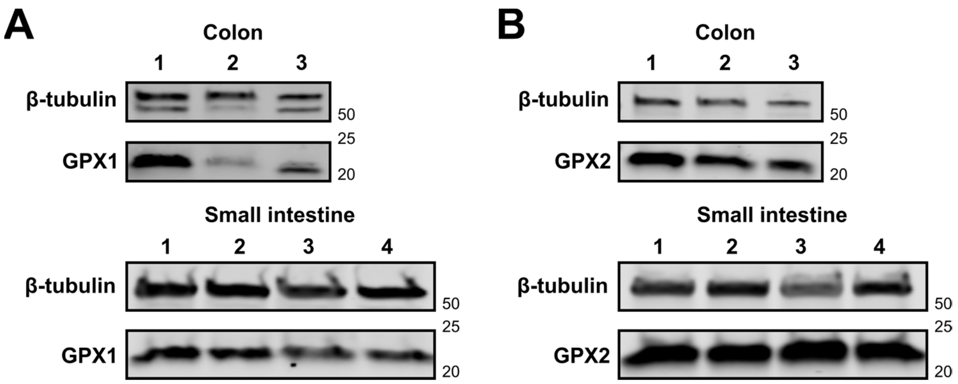


**Figure S1. GPX1 and GPX2 protein expression in WT mouse colon and small intestine epithelium.** Western blots for (A) GPX1, (B) GPX2, and (A, B) β-tubulin (loading control) in WT mouse colon and small intestine epithelium. n=3-4 mice.

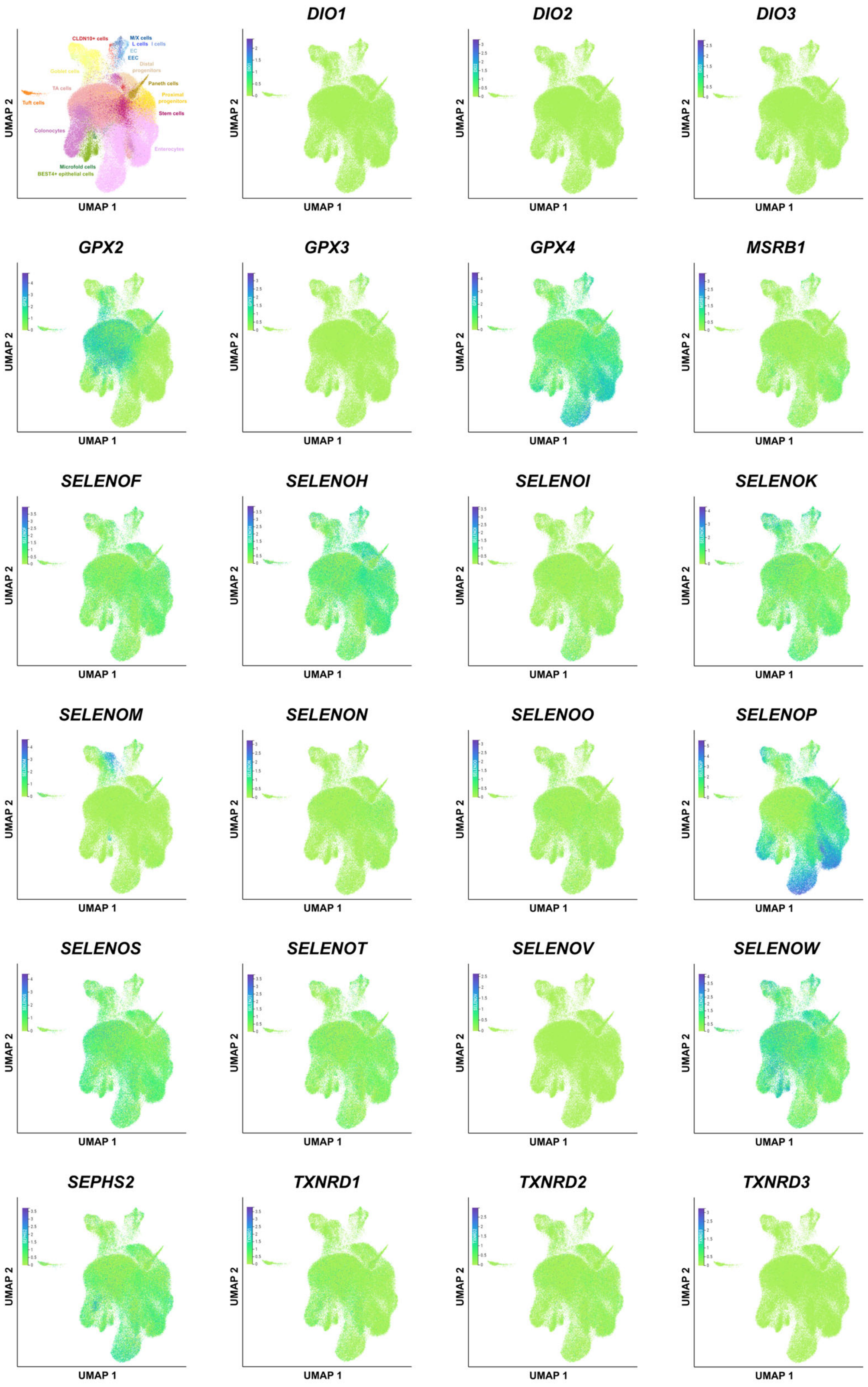


**Figure S2. Selenoprotein expression in the normal human colon and small intestine.** Gut Cell Atlas scRNA-seq data from human colon and small intestine epithelium queried for indicated selenoproteins. EC: enterochromaffin, EEC: enteroendocrine, TA: transit amplifying. n=6 donors.

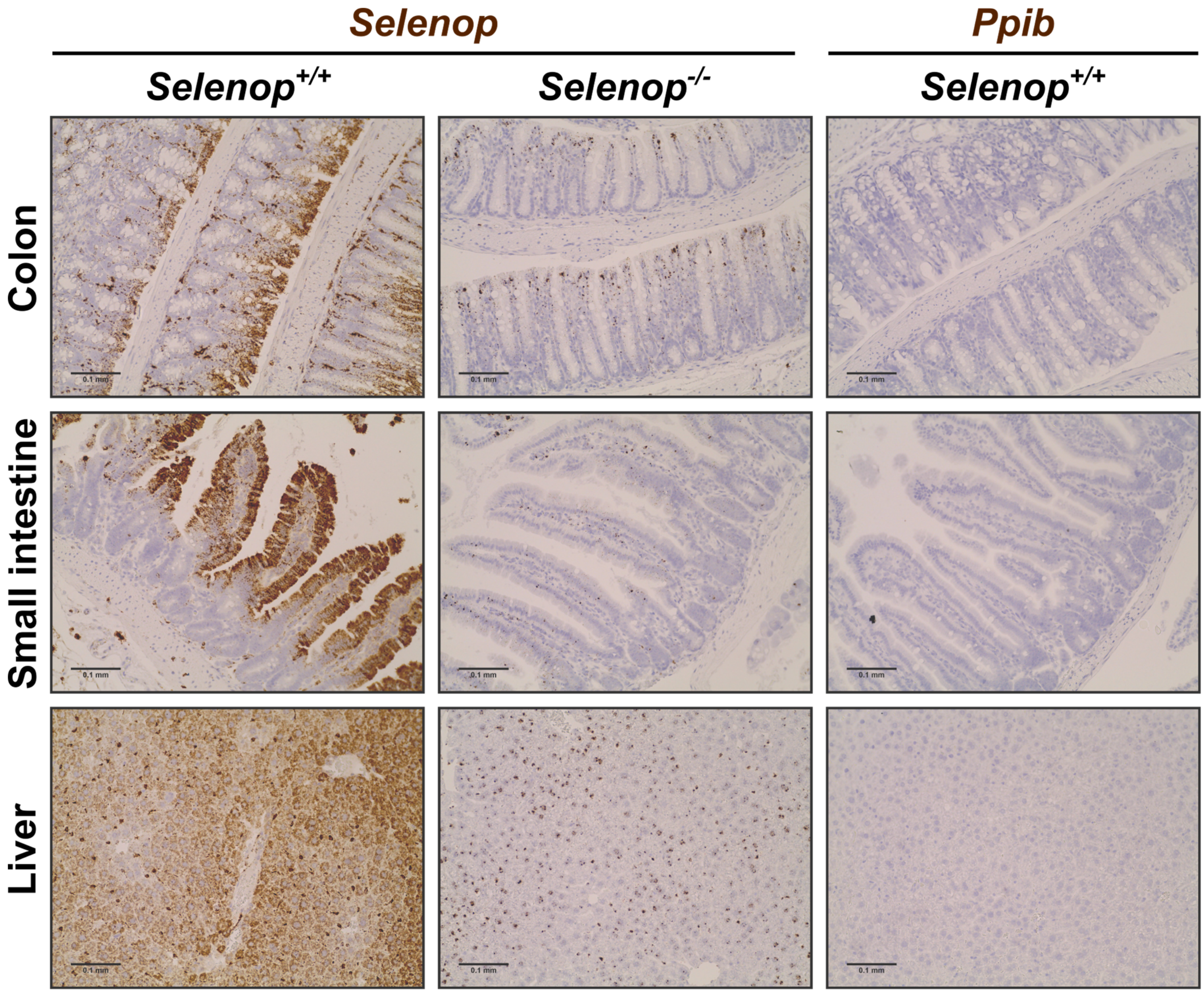


**Figure S3. Validation of *Selenop* RNAscope® probe**. RNAscope® of *Selenop*$^{+/+}$ and *Selenop*$^{-/-}$ colon, small intestine, and liver for *Selenop* or *Ppib* (negative control). Representative 20x images, scale bars = 100 µm.

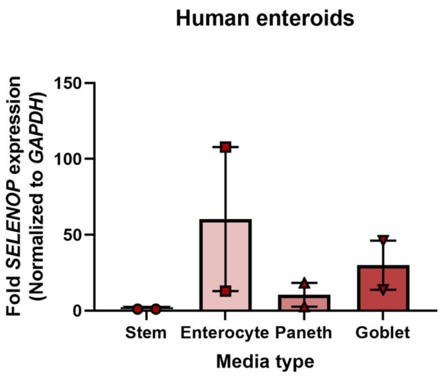


**Figure S4.** ***SELENOP*** **expression in differentiated human enteroids.** RT-qPCR for *SELENOP* of human enteroids subjected to directed differentiation protocols. Pooled data from n=2 independent experiments. Data are displayed as mean ± SEM.

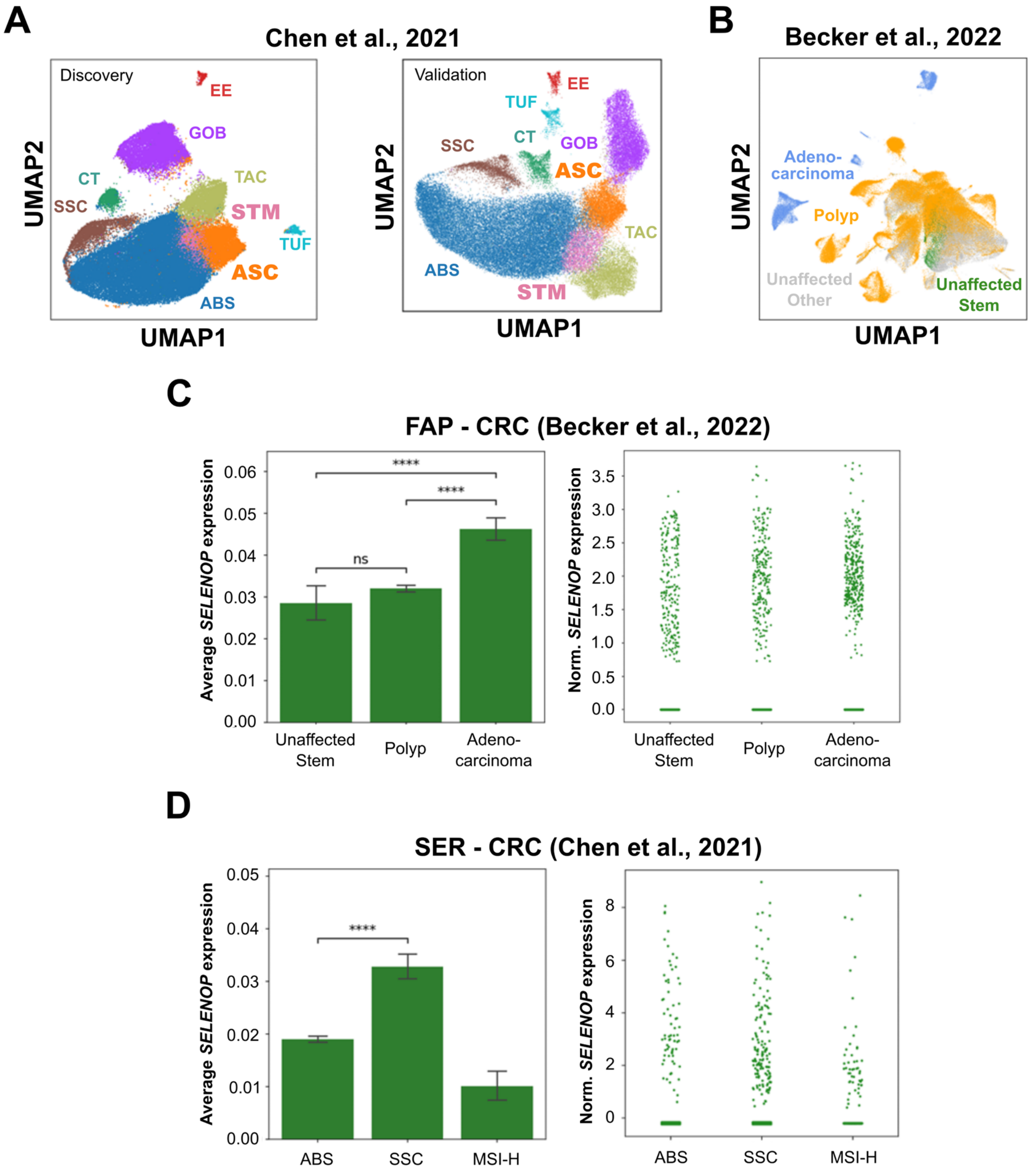


**Figure S5. *SELENOP* expression throughout colorectal cancer progression.** (A) scRNA-seq data from human colorectal polyps and normal colon tissue. (Left) Discovery cohort: n=35 normal samples, n=27 polyps, n=70,691 cells. (Right) Validation cohort: n=31 normal samples, n=28 polyps, n=71,374 cells. ABS: absorptive, ASC: adenoma-specific cells, CT: crypt top, EE: enteroendocrine, GOB: goblet, STM: stem, SSC: serrated-specific cells, TAC: transit amplifying cells, TUF: tuft. (B, C) snRNA-seq data from human colorectal polyps/cancers and normal colon tissue. n=23 normal samples, n=42 polyps, n=5 cancers, n=161,809 cells. (C) *SELENOP* expression by cell type. CRC: colorectal cancer, FAP: familial adenomatous polyposis. (D) scRNA-seq data from human colorectal polyps/cancers and normal colon tissue. *SELENOP* expression by cell type. ABS: absorptive, MSI-H: microsatellite instability-high, SER: serrated polyp, SSC: serrated-specific cells. n=21 normal samples, n=19 polyps, n=2 cancers. Kruskal-Wallis tests with 2-sided Mann-Whitney tests. ****p<0.0001. Data are displayed as mean ± SD.

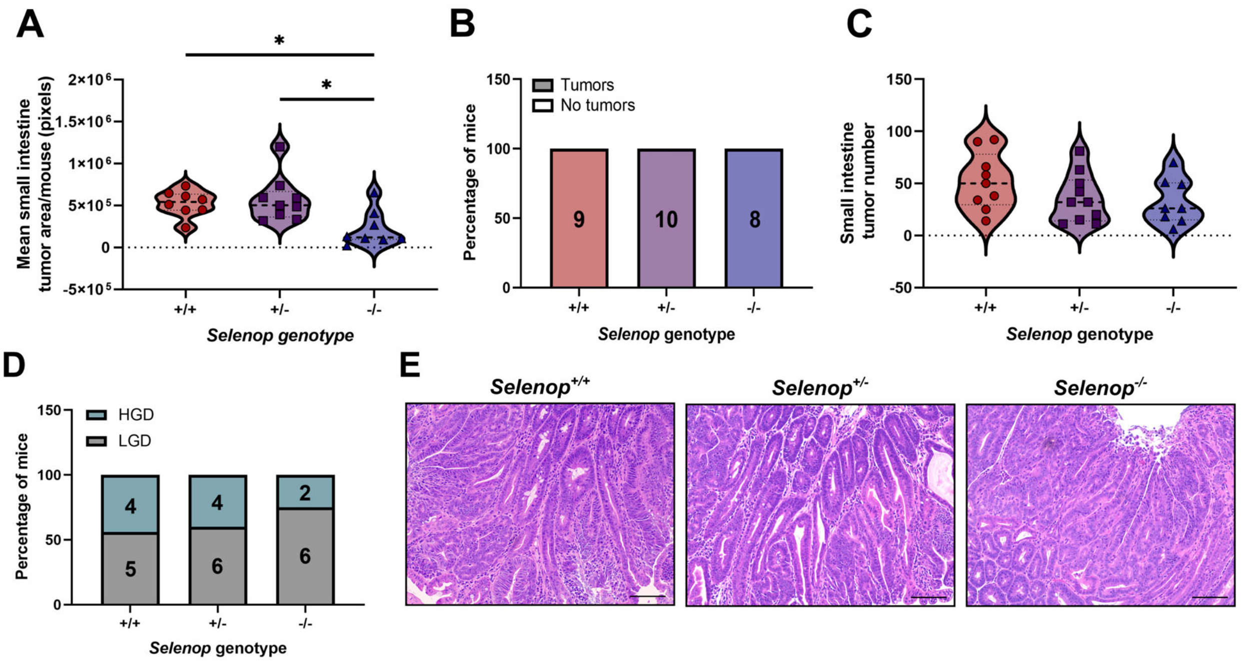


**Figure S6. *Selenop* KO decreases small intestine tumor size in *Apc*-dependent tumorigenesis.** (A) Small intestine tumor area, (B) small intestine tumor incidence, (C) small intestine tumor number, (D) small intestine tumor dysplasia scores (HGD: high-grade dysplasia, LGD: low-grade dysplasia), and (E) small intestine tumor histology of $Apc^{\Delta IE/+}$; $Selenop^{+/+}$ (n=9), $Selenop^{+/-}$ (n=10), and $Selenop^{-/-}$ (n=8) mice. Pooled data from n=2 independent experiments. Representative 20x images (E), scale bars = 100 µm. Kruskal-Wallis tests (A, C), Freeman-Halton tests (B, D). *p<0.05.

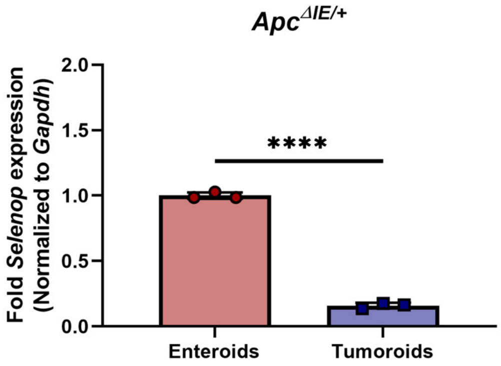


**Figure S7. *Selenop* expression is reduced in tumoroids.** RT-qPCR for *Selenop* of *Apc*$^{\Delta IE/+}$; *Selenop*$^{+/+}$ enteroids and tumoroids. Pooled data from n=3 mice. 2-sided unpaired t test. ****$p<0.0001$. Data are displayed as mean ± SD.

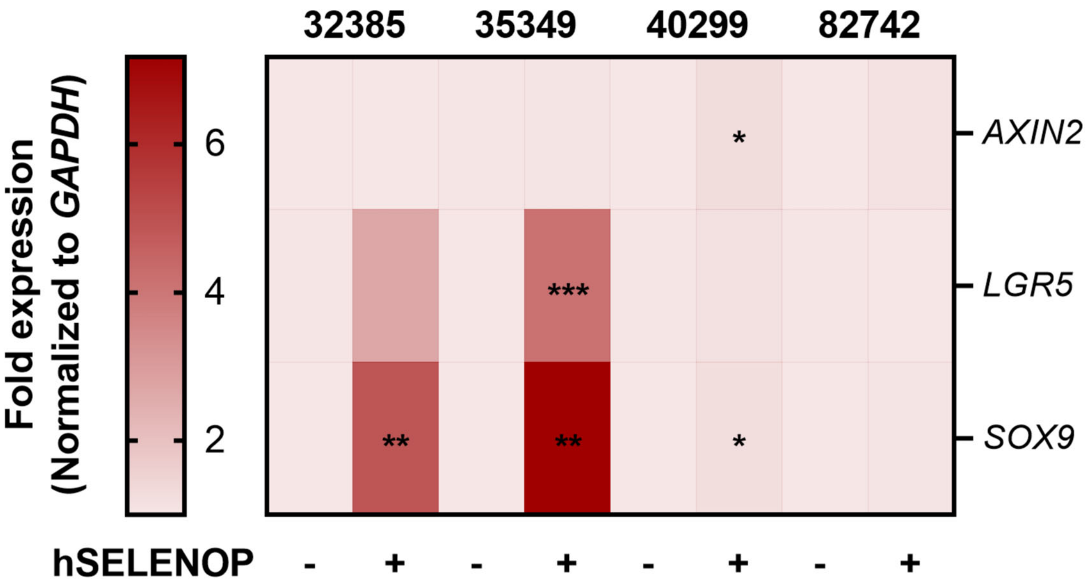


**Figure S8. SELENOP increases WNT target gene expression in human tumoroids.** RT-qPCR for *AXIN2*, *LGR5*, and *SOX9* of human tumoroids. Each five-digit number represents tumoroids established from one patient. Pooled data from n=3 independent experiments. 2-sided paired t tests. *p<0.05, **p<0.01, ***p<0.001. Data are displayed as mean.

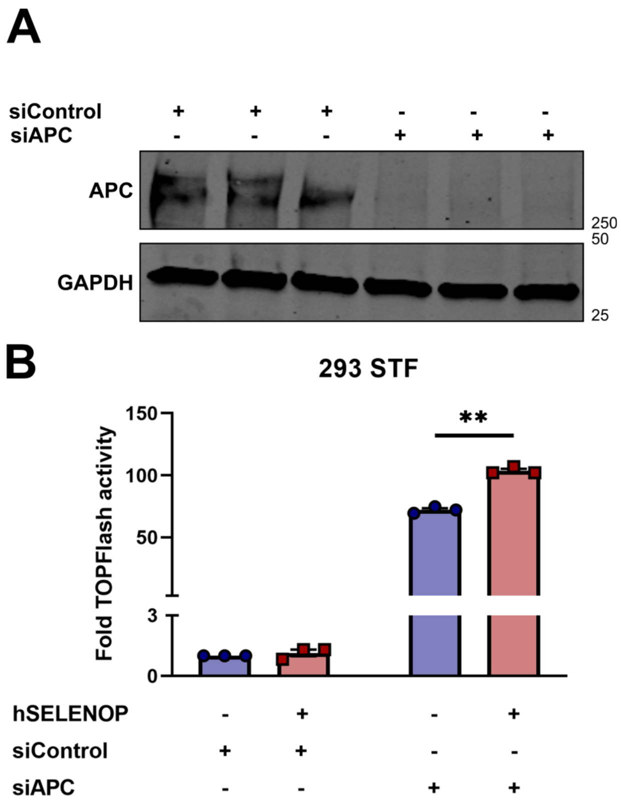


**Figure S9. SELENOP acts upstream of APC.** (A) Western blot for APC and GAPDH (loading control) of lysates from 293 STF cells transfected with siControl or siAPC. (B) TOPFlash activity of 293 STF cells transfected with siControl or siAPC and treated without or with hSELENOP. Pooled data from n=3 independent experiments. 2-way repeated measures ANOVA with 2-sided Sidak's multiple comparisons test. **$p<0.01$. Data are displayed as mean ± SEM.

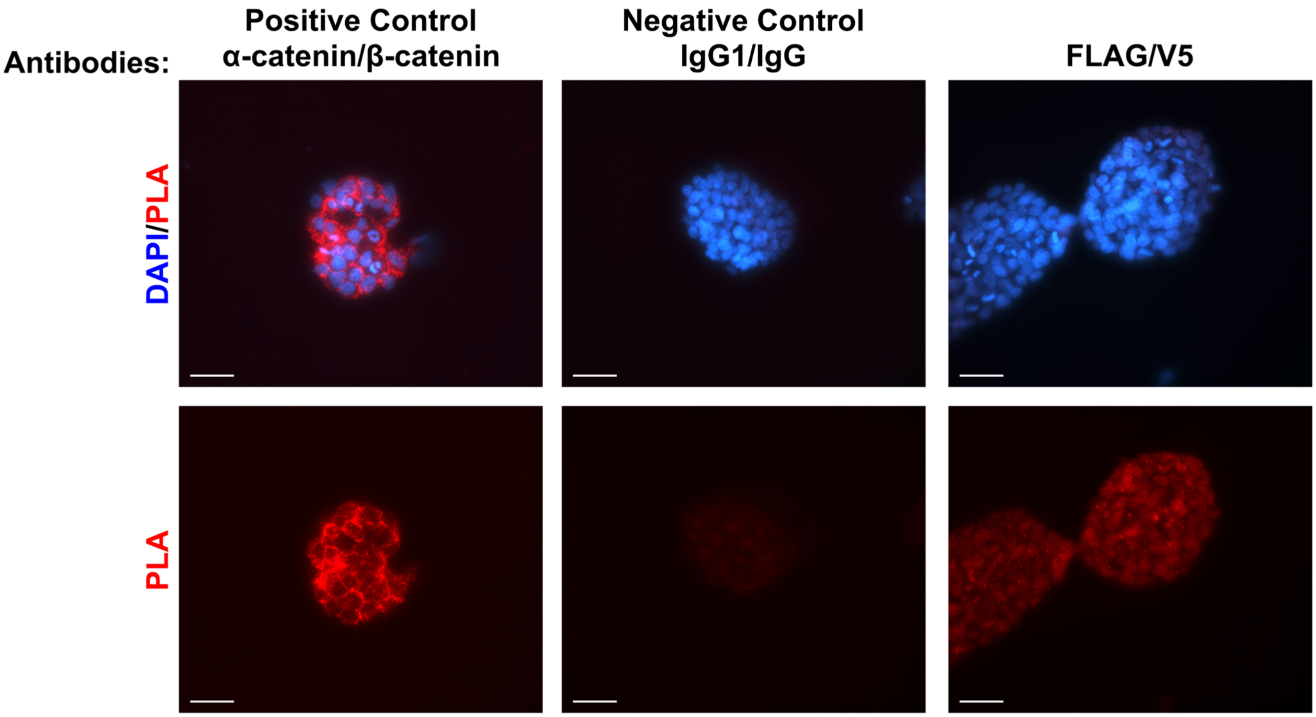


**Figure S10. SELENOP interacts with LRP6.** Proximity ligation assay of 293T cells co-transfected with FLAG-mLRP6 and V5-mSELENOP. Representative 40x images from n=3 independent experiments, scale bars = 50 µm.

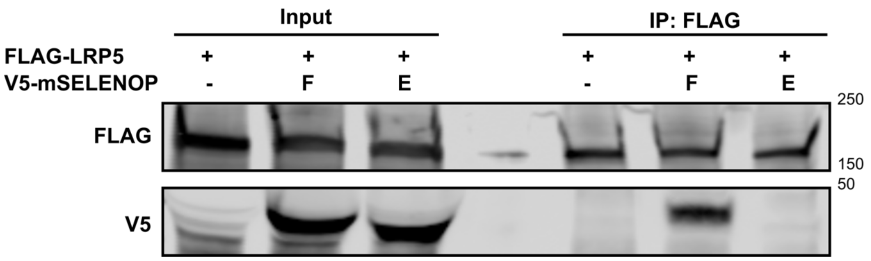


**Figure S11. SELENOP$^{U258\text{-}U299}$ mediates the SELENOP:LRP5 interaction.** Western blot for FLAG and V5 of FLAG IPs from 293T cells co-transfected with FLAG-mLRP5 and full-length (F) or LRP5/6-uncoupling (E) V5-mSELENOP. Representative data from n=2 independent experiments.

## Full unedited blot for Figure 7A

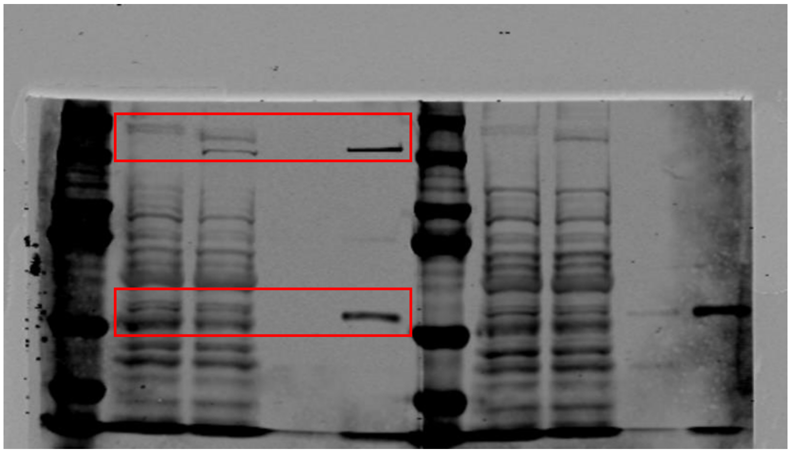

Mouse anti-FLAG (F1804, Sigma-Aldrich)

Mouse anti-SELENOP (N11, VAPR)

## Full unedited blot for Figure 7B

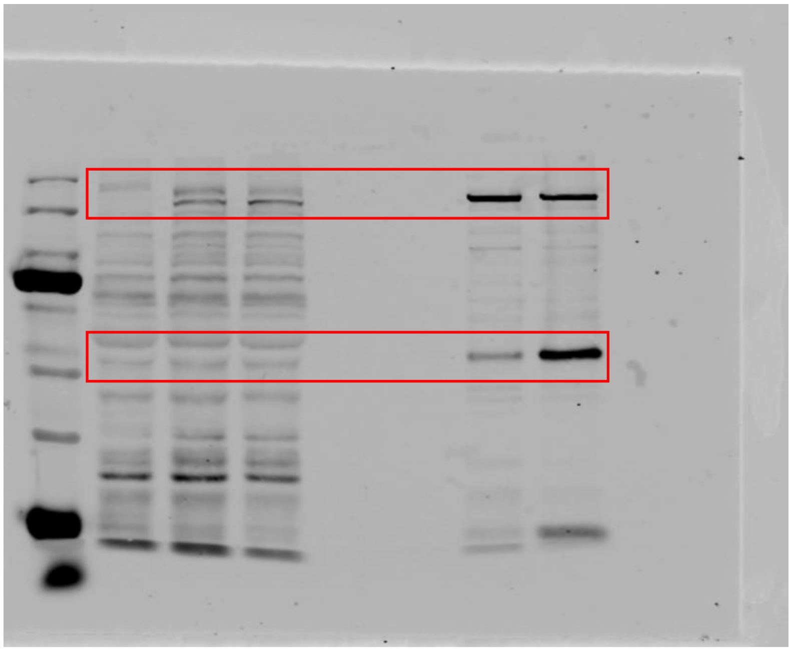

Mouse anti-FLAG (F1804, Sigma-Aldrich)

Mouse anti-SELENOP (N11, VAPR)

## Full unedited blot for Figure 7C

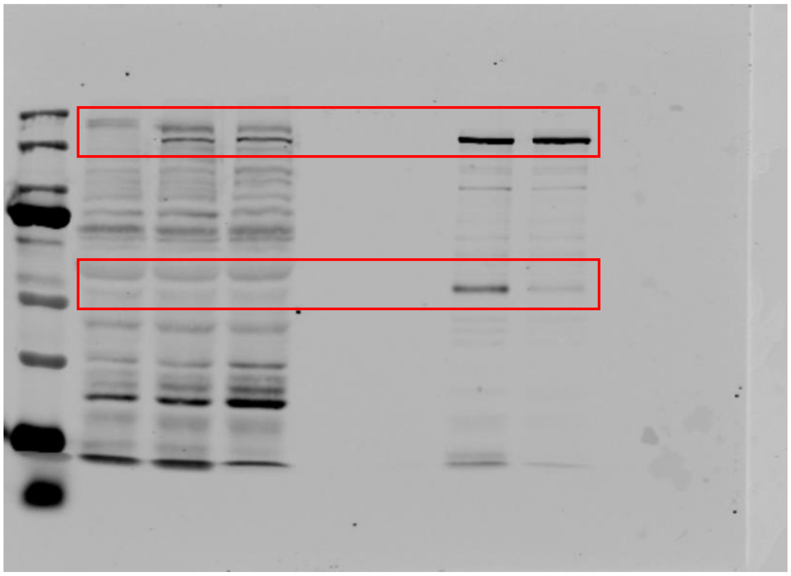

Mouse anti-FLAG (F1804, Sigma-Aldrich)

Mouse anti-SELENOP (N11, VAPR)

# Full unedited blot for Figure 7D

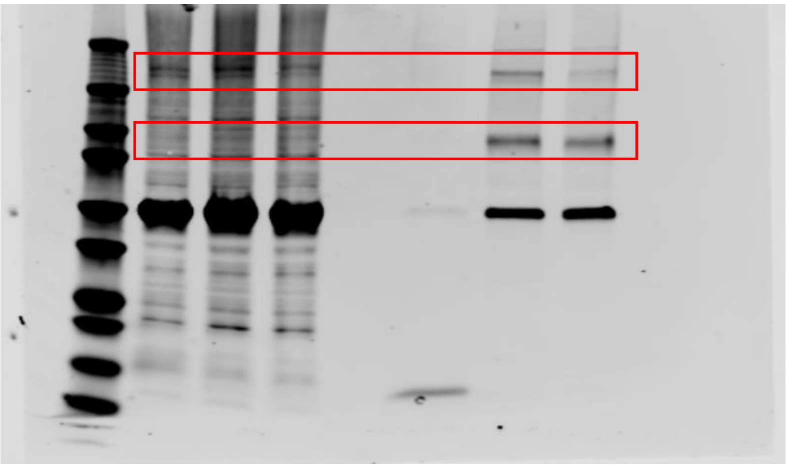

Rabbit anti-LRP6 (2560, Cell Signaling Technology)

Rabbit anti-Na$^+$/K$^+$-ATPase (3010, Cell Signaling Technology)

Rabbit anti-β-tubulin (2146, Cell Signaling Technology)

Same blot, different contrast/brightness settings on Odyssey

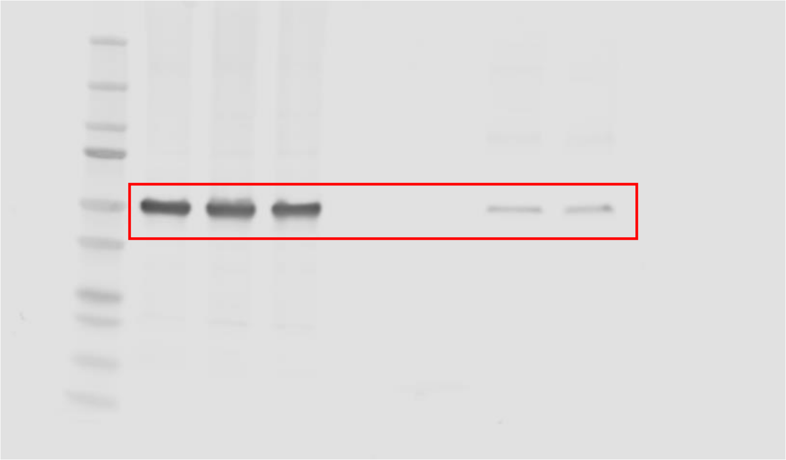

Rabbit anti-LRP6 (2560, Cell Signaling Technology)

Rabbit anti-Na$^+$/K$^+$-ATPase (3010, Cell Signaling Technology)

Rabbit anti-β-tubulin (2146, Cell Signaling Technology)

Same blot, quantified on Odyssey

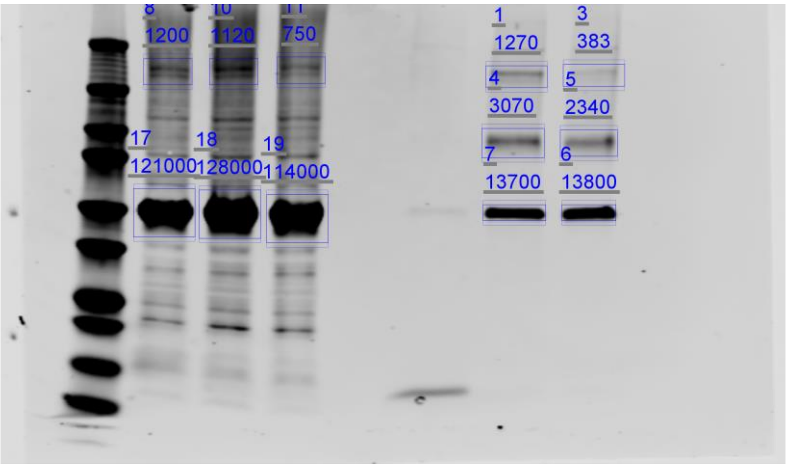


Rabbit anti-LRP6 (2560, Cell Signaling Technology)

Rabbit anti-Na$^+$/K$^+$-ATPase (3010, Cell Signaling Technology)

Rabbit anti-β-tubulin (2146, Cell Signaling Technology)

# Full unedited blot for Figure 8B

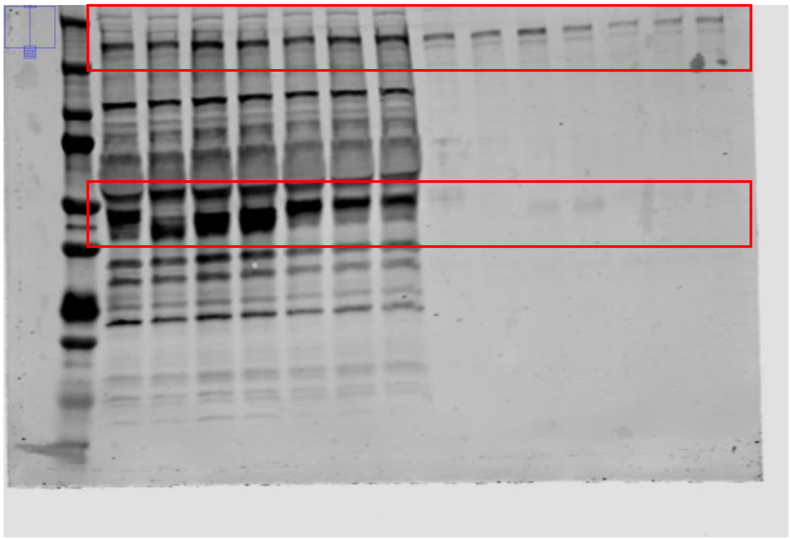

Rabbit anti-LRP6 (3395, Cell Signaling Technology)

Rabbit anti-SELENOP (Proteintech Group)

Same blot, different contrast/brightness settings on Odyssey

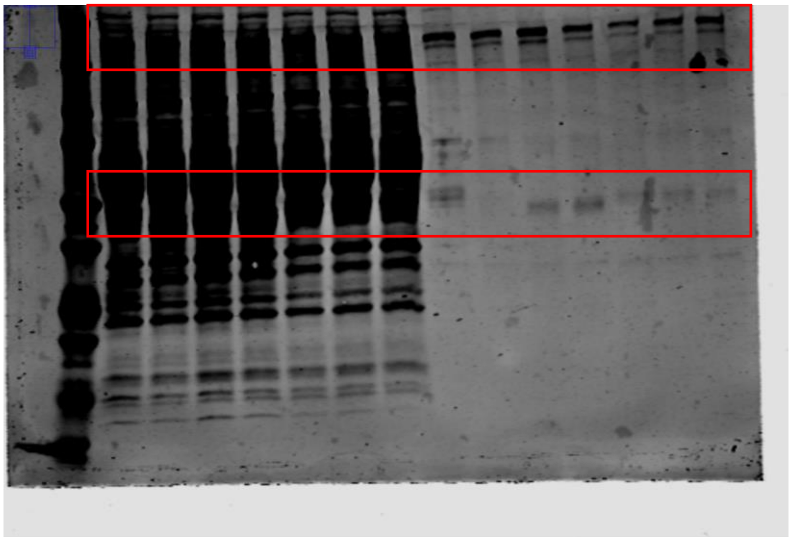

Rabbit anti-LRP6 (3395, Cell Signaling Technology)

Rabbit anti-SELENOP (Proteintech Group)

# Full unedited blot for Figure 8D

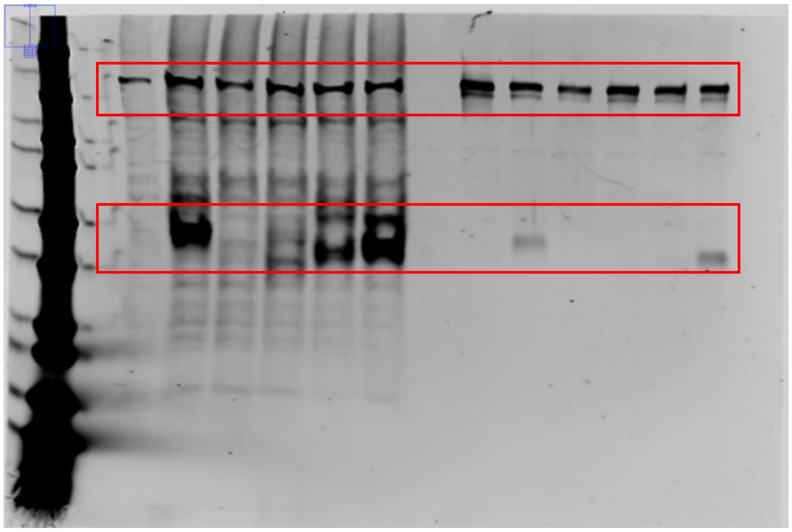

Rabbit anti-LRP6 (3395, Cell Signaling Technology)

Rabbit anti-V5 (13202, Cell Signaling Technology)

## Full unedited blot for Figure 9B

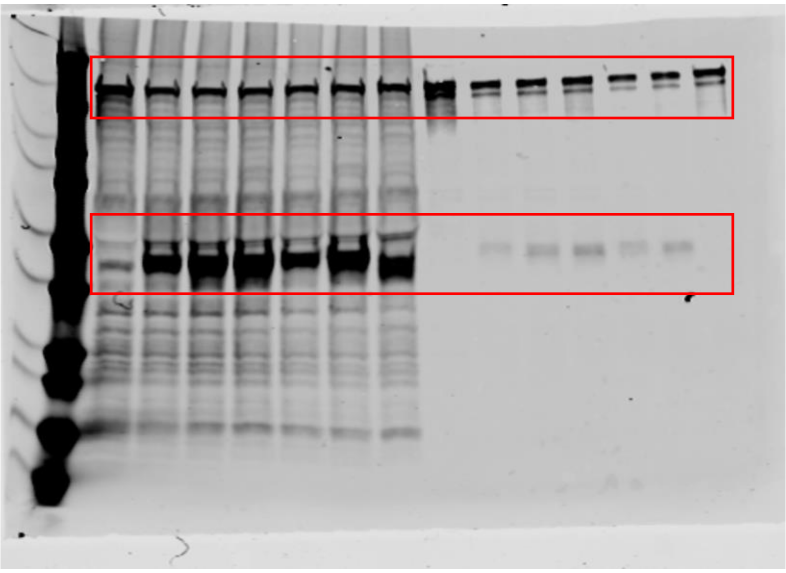

Rabbit anti-LRP6 (3395, Cell Signaling Technology)

Rabbit anti-V5 (13202, Cell Signaling Technology)

## Full unedited blot for Figure 9C

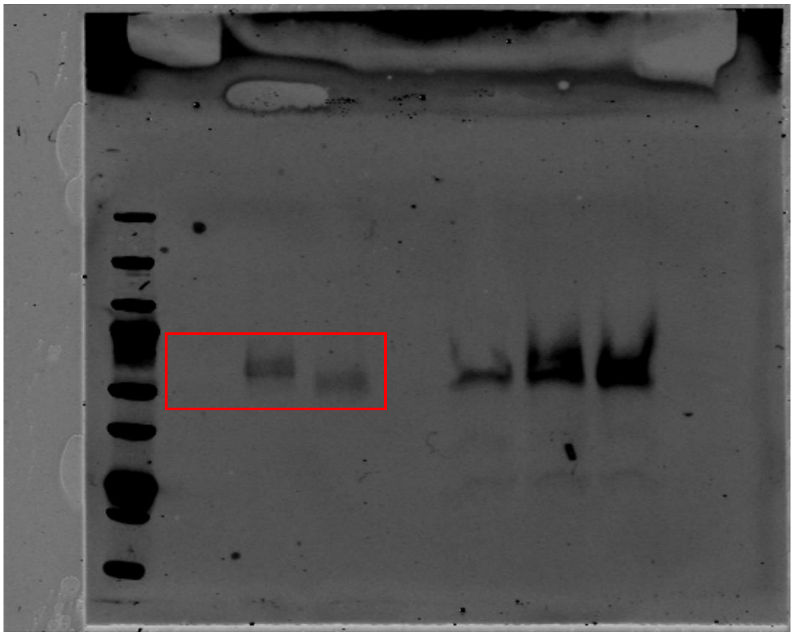

Mouse anti-V5 (ab27671, abcam)

# Full unedited blot for Figure S1A

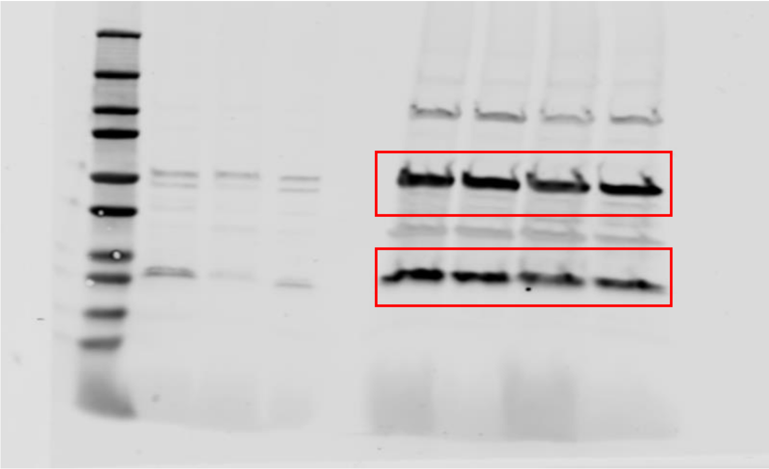

Rabbit anti-β-tubulin (2146, Cell Signaling Technology)

Rabbit anti-GPX1 (SAB2700534, Sigma-Aldrich)

Same blot, different contrast/brightness settings on Odyssey

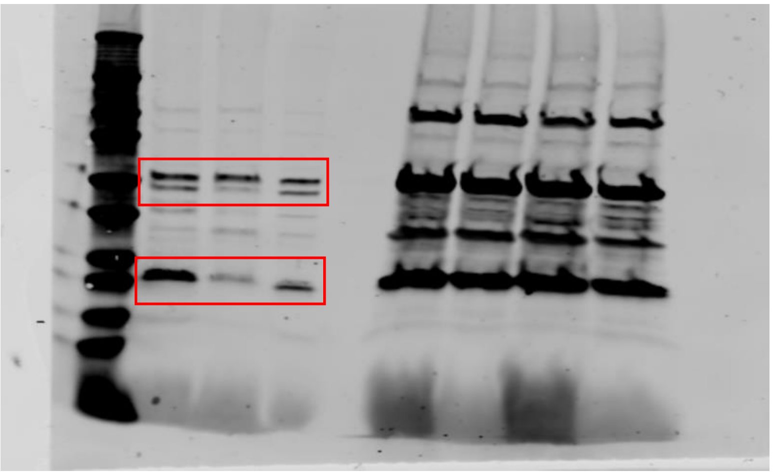

Rabbit anti-β-tubulin (2146, Cell Signaling Technology)

Rabbit anti-GPX1 (SAB2700534, Sigma-Aldrich)

# Full unedited blot for Figure S1B

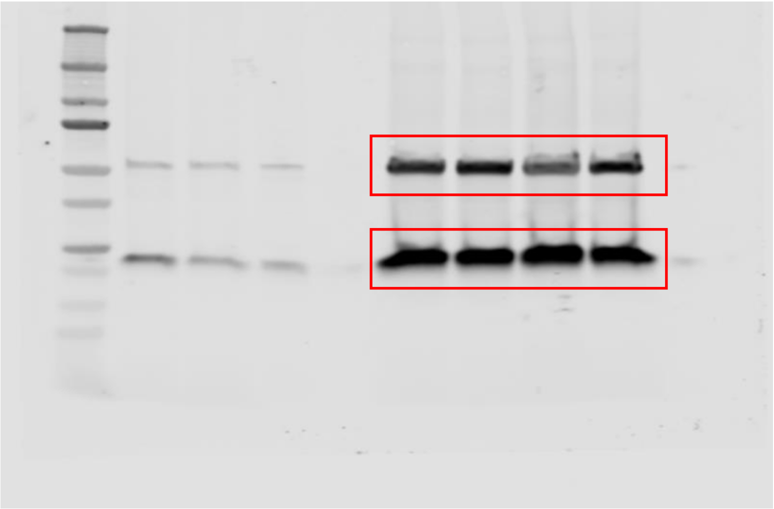

Rabbit anti-β-tubulin (2146, Cell Signaling Technology)

Rabbit anti-GPX2 (ab137431, abcam)

Same blot, different contrast/brightness settings on Odyssey

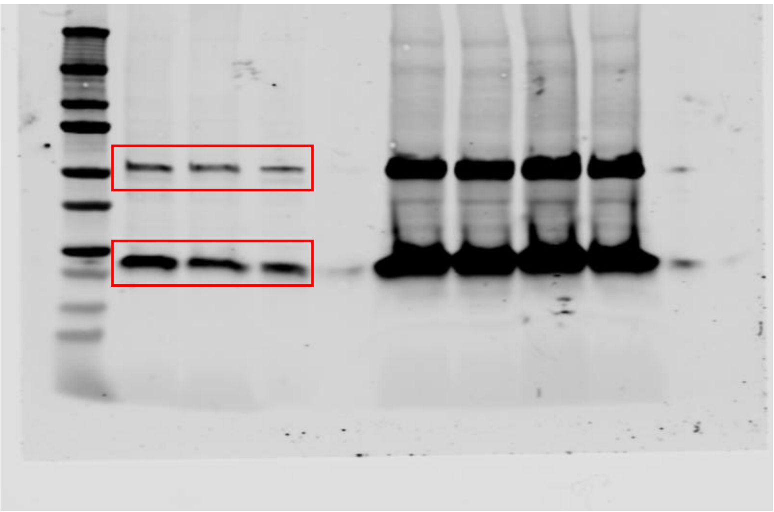

Rabbit anti-β-tubulin (2146, Cell Signaling Technology)

Rabbit anti-GPX2 (ab137431, abcam)

# Full unedited blot for Figure S9A

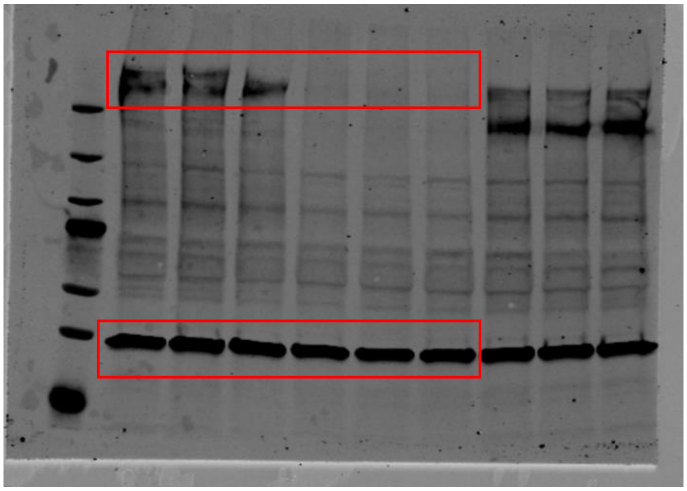

Rabbit anti-APC (sc-7930, Santa Cruz Biotechnology)

Rabbit anti-GAPDH (5174, Cell Signaling Technology)

# Full unedited blot for Figure S11

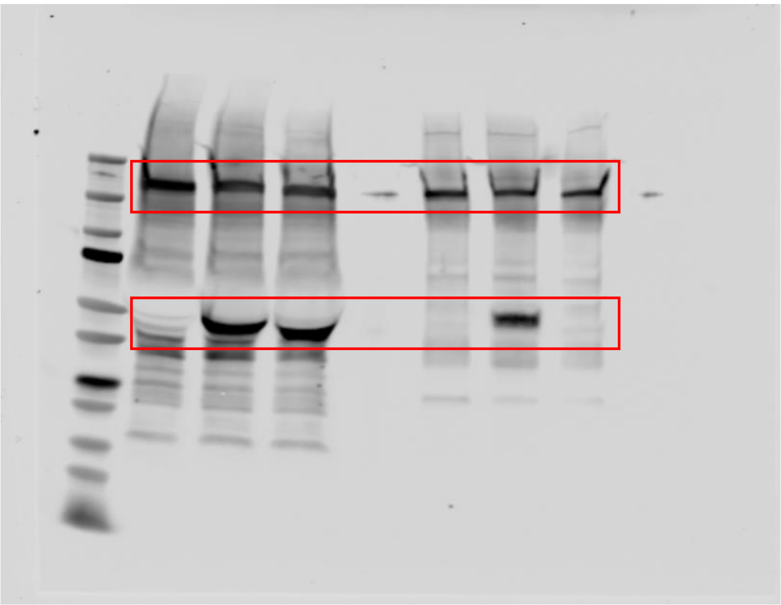

Mouse anti-FLAG (F1804, Sigma-Aldrich)

Rabbit anti-V5 (13202, Cell Signaling Technology)
